# Fungus-derived protein particles as cell-adhesive matrices for cell-cultivated food

**DOI:** 10.1038/s41538-023-00209-y

**Published:** 2023-07-13

**Authors:** Yu Xing Teo, Kah Yin Lee, Corinna Jie Hui Goh, Loo Chien Wang, Radoslaw M. Sobota, Keng-Hwee Chiam, Chan Du, Andrew C. A. Wan

**Affiliations:** 1 Singapore Institute of Food and Biotechnology Innovation, Agency for Science, Technology, and Research (A*STAR), Singapore, 138669 Singapore; 2 Bioinformatics Institute, Agency for Science, Technology, and Research (A*STAR), Singapore, 138671 Singapore; 3 Functional Proteomics Laboratory, SingMass National Laboratory, Institute of Molecular and Cell Biology, Agency for Science, Technology, and Research (A*STAR), Singapore, 138673 Singapore

**Keywords:** Cell adhesion, Nutrition

## Abstract

Cell-adhesive factors mediate adhesion of cells to substrates via peptide motifs such as the Arg–Gly–Asp (RGD) sequence. With the onset of sustainability issues, there is a pressing need to find alternatives to animal-derived cell-adhesive factors, especially for cell-cultivated food applications. In this paper, we show how data mining can be a powerful approach toward identifying fungal-derived cell-adhesive proteins and present a method to isolate and utilize these proteins as extracellular matrices (ECM) to support cell adhesion and culture in 3D. Screening of a protein database for fungal and plant proteins uncovered that ~5.5% of the unique reported proteins contain RGD sequences. A plot of fungi species vs RGD percentage revealed that 98% of the species exhibited an RGD percentage > = 1%. We observed the formation of protein particles in crude extracts isolated from basidiomycete fungi, which could be correlated to their stability towards particle aggregation at different temperatures. These protein particles were incorporated in 3D fiber matrices encapsulating mouse myoblast cells, showing a positive effect on cell alignment. We demonstrated a cell traction stress on the protein particles (from *Flammulina velutipes*) that was comparable to cells on fibronectin. A snapshot of the RGD-containing proteins in the fungal extracts was obtained by combining SDS-PAGE and mass spectrometry of the peptide fragments obtained by enzymatic cleavage. Therefore, a sustainable source of cell-adhesive proteins is widely available in the fungi kingdom. A method has been developed to identify candidate species and produce cell-adhesive matrices, applicable to the cell-cultivated food and healthcare industries.

## Introduction

Cell-adhesive materials, specifically materials that can support the attachment, spreading, proliferation, and differentiation of cells are widely used in the biomedical and pharmaceutical industries. In tissue engineering, for example, such materials are important to mediate adhesion of various cell types to scaffolds for tissue or organ regeneration^1^. The common biomaterials used for mammalian cell adhesion are animal-derived extracellular proteins such as collagen and fibronectin. They have the advantage of being biocompatible, in addition to possessing native ligands that can be recognized and bound by a wide range of mammalian cell types.

In recent years, sustainability and animal welfare concerns have given rise to an endeavour to replace animal products with those obtained from cells grown in culture e.g., cell-cultivated meat, milk, and liver^2^. Some of the cell types employed in these nascent industries, such as muscle cells, are anchorage-dependent, requiring adhesion to a culture support material in order to survive and proliferate. Ideally, these cell culture support materials are non-animal-derived and edible, such that they do not need to be dissociated from the propagated cells and form part of the food product, thus saving time and cost.

One approach toward identifying potential cell-adhesive proteins in nature would be to look for proteins that contain the Arg–Gly–Asp (RGD) motif, which was the first cell-adhesive peptide sequence to be identified and remains the most widely applied^1,3^. A literature search turns up no more than a few reports on RGD-containing plant proteins, such as the renin-like protein in the cardoon, *Cynara cardunculus*^4^. In the fungal kingdom, more literature on RGD-containing proteins is available, particularly as they are found quite widely in pathogenic fungi^5–7^.

## Results

### Screening for RGD-containing proteins in fungi and plantae kingdom

**Fig. 1:**
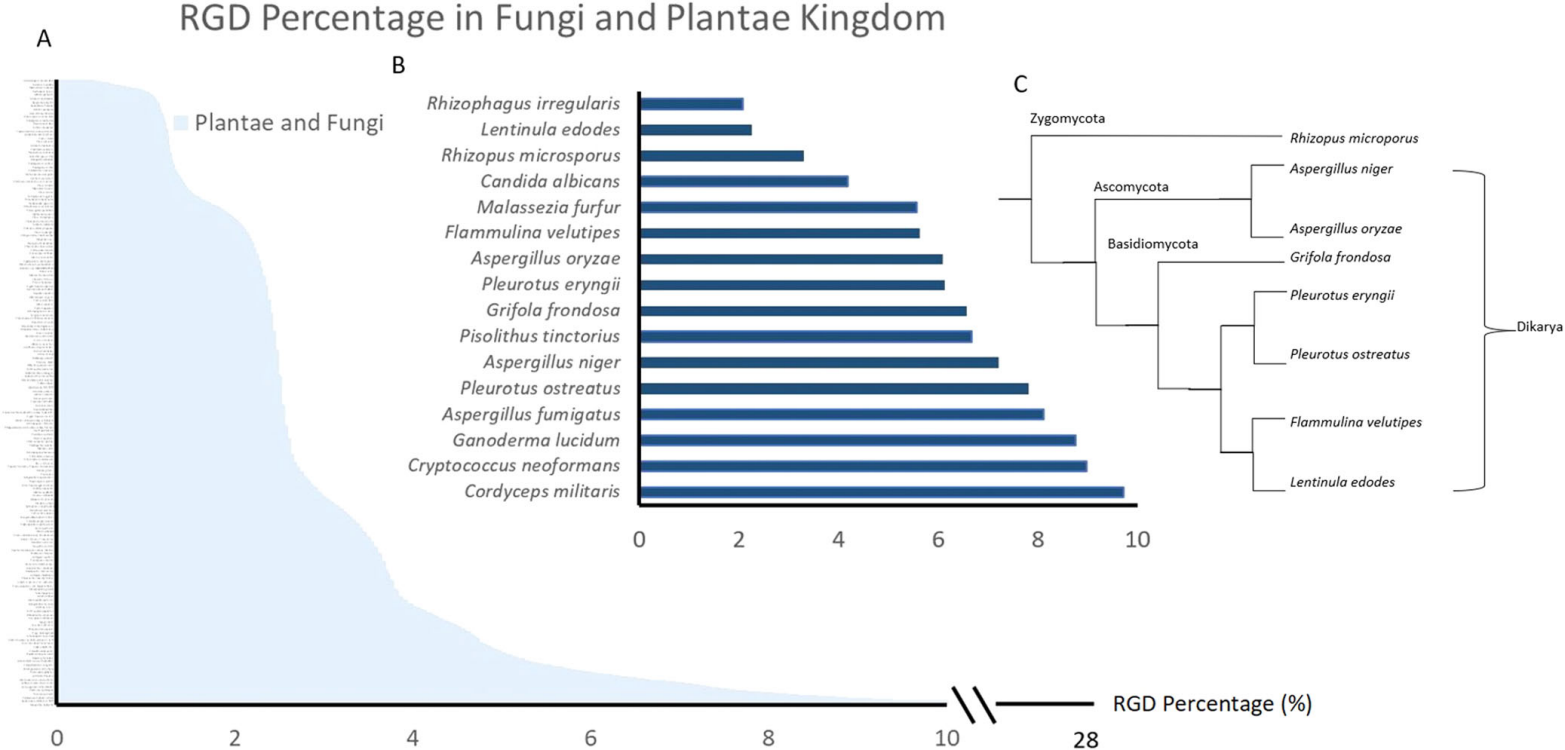
RGD percentage and phylogenetic relationship of edible and parasitic fungi. **A** RGD percentage in the fungi and plant kingdom; **B** parasitic and edible fungi are represented by the blue bars. **C** Phylogenetic tree showing the relationship between some of the fungi in (**B**).

We carried out a systematic search on RGD-containing proteins by screening for all the unique RGD-containing protein sequences in the GenBank database. Out of a total of 6,346,526 proteins reported in the database, 352,056 of the proteins contained an RGD sequence, corresponding to about 5.5%. We proceeded to define an RGD percentage for each species in the database: RGD% = (number of reported proteins containing RGD/total number of reported proteins in the species) × 100. This is represented by the horizontal axis of Fig. 1A. Interestingly, ~98% of all fungal and plant species possess an RGD percentage of greater than 1%, and about 8.8% of the species had an RGD% of greater than 5%. This means that for a large majority of fungi/plants, more than 1 out of 100 proteins reported contain the RGD sequence, indicating that the motif is more widespread than commonly perceived. Another surprising fact was the high RGD percentage observed for the plant kingdom. Upon further analysis, this was found to be mainly due to the presence of the RGD motif in the chloroplast protein, petG^8^. However, as a large fraction of the RGD-containing proteins in the plant kingdom remains unmapped in terms of function^9^, we chose to focus on the fungal kingdom instead.

Representative species of parasitic and edible fungi are indicated in Fig. 1B. While edible fungi are mainly the fruiting bodies of saprophytic species, we also included molds such as *Aspergillus oryzae* and *Rhizopus microsporus* that are used in food fermentation. From the plot, several species of parasitic fungi exhibited the highest RGD percentages of between 8 and 10%, namely *Aspergillus fumigatus*, *Ganoderma lucidum*, *Cryptococcus Neoformans* and *Cordyceps militaris*. However, the common, edible species also exhibited RGD percentages of between 2 and 8%. Amongst these were three species from the order Basidiomycota, namely *Lentinus edodes, Flammulina velutipes and Pleurotus eryngii*, that we selected for further experimentation. These are fruiting bodies (mushrooms) that are easily obtained from retail.

### Precipitation and analysis of fungal extracts

**Fig. 2:**
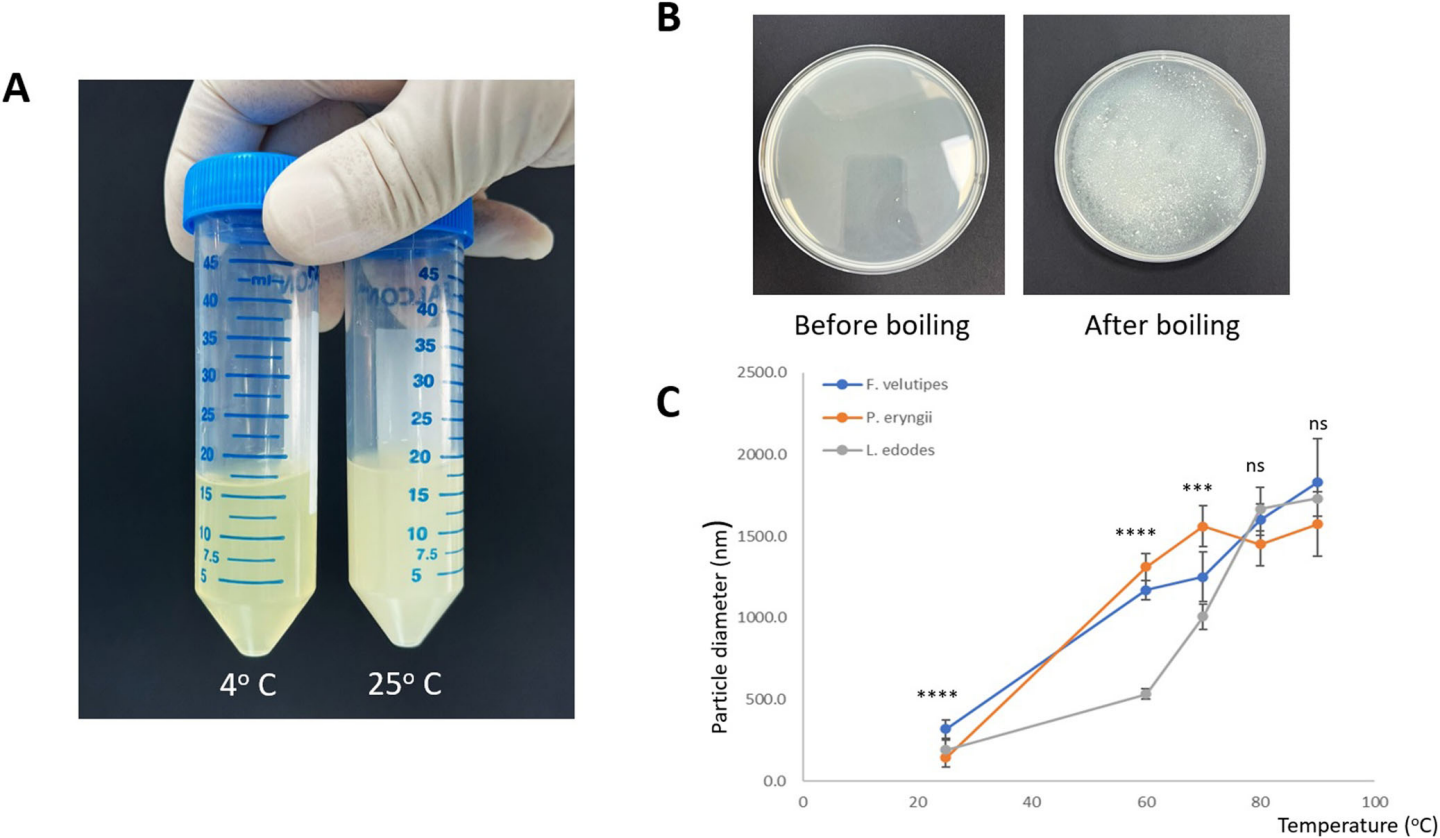
Precipitation and analysis of fungal extracts. **A** Comparison of crude extracts from *F. velutipes* after standing at 4 °C and room temperature (25 °C) for 4 days. For the extract at 25 °C, spontaneous precipitation was observed to give a suspension of white particles. **B** Boiling of the protein extract accelerated the formation of the particles. **C** Growth of particle diameters at increasing temperature for extracts from the fungal species, reflecting their respective stability towards protein aggregation. ANOVA tests were performed to compare the particle diameter between different fungi at each particular temperature. The error bars represent one standard deviation of uncertainty. The *P* values are represented by asterisks (*); *P ≤ 0.05; **P ≤ 0.01; ***P ≤ 0.001; ****P ≤ 0.0001; ns: *P* > 0.05.

Crude extracts were obtained from two of these species (*F. velutipes* and *L. edodes*) for preliminary determination of cell-adhesive properties. The extracts were used to coat plates with high absorption affinity for proteins and seeded with MCF7 cell line and primary skeletal muscle cells, respectively. The results indicated that proteins were present in the crude extracts that enhanced cell adhesion (Supplementary Fig. 1). At this juncture, two options were available for the development of a cell culture matrix from the fungal proteins. The first involved isolating the individual proteins and using them individually or in combination. The second alternative was to find a means to reconstitute them into an insoluble fraction with cell-adhesive function, an option which we deemed much more straightforward and inexpensive. A serendipitous observation provided a possible method by which this could be achieved. When the crude extracts were allowed to stand at room temperature, the spontaneous formation of a white precipitate was observed to occur (Fig. 2A). The rate of precipitation was starkly different for the three fungal species- for *P. eryngii*, precipitation occurred in a matter of hours, for *F. velutipes* within 1–2 days, while the crude extract for *L. edodes* remained stable for weeks before slight precipitation was observed.

It was suspected that denaturation of protein, leading to aggregation was responsible for the precipitation. If denaturation was responsible for protein particle formation, it followed that heating of the crude extract would speed up the process. This was borne out by experiment (Fig. 2B). Particle diameters were measured for *F. velutipes*, *P. eryngii*, and *L. edodes* at different temperatures, showing that protein aggregation occurred with increasing temperature for all cases, with a precipitation rate in the order *P. eryngii* > *F. velutipes* > *L. edodes*, as expected from their respective stability towards aggregation (Fig. 2C). This order of precipitation was not due to a difference in protein concentration in the crude extracts of the different fungi, as L. Edodes, with the highest crude extract protein concentration, precipitated at the slowest rate, whereas *P. eryngii*, with the lowest crude extract protein concentration, precipitated at the fastest rate (Fig. 2C and Supplementary Fig. 2–1).

As reflected in the plot, the protein particles possessed diameters in the range of 146–1831 nm. A model was established for particle aggregation dynamics, to elucidate the relationship between particle size and the various conditions of temperature and fungal biomass concentration employed, as described in the Supplementary Fig. 2–2 and 2–3. For the case of *L. edodes*, while aggregation increased at higher temperatures, there was no clear dependence of aggregation on fungal biomass concentration.

### Cell-alignment and cell-adhesion analysis

**Fig. 3:**
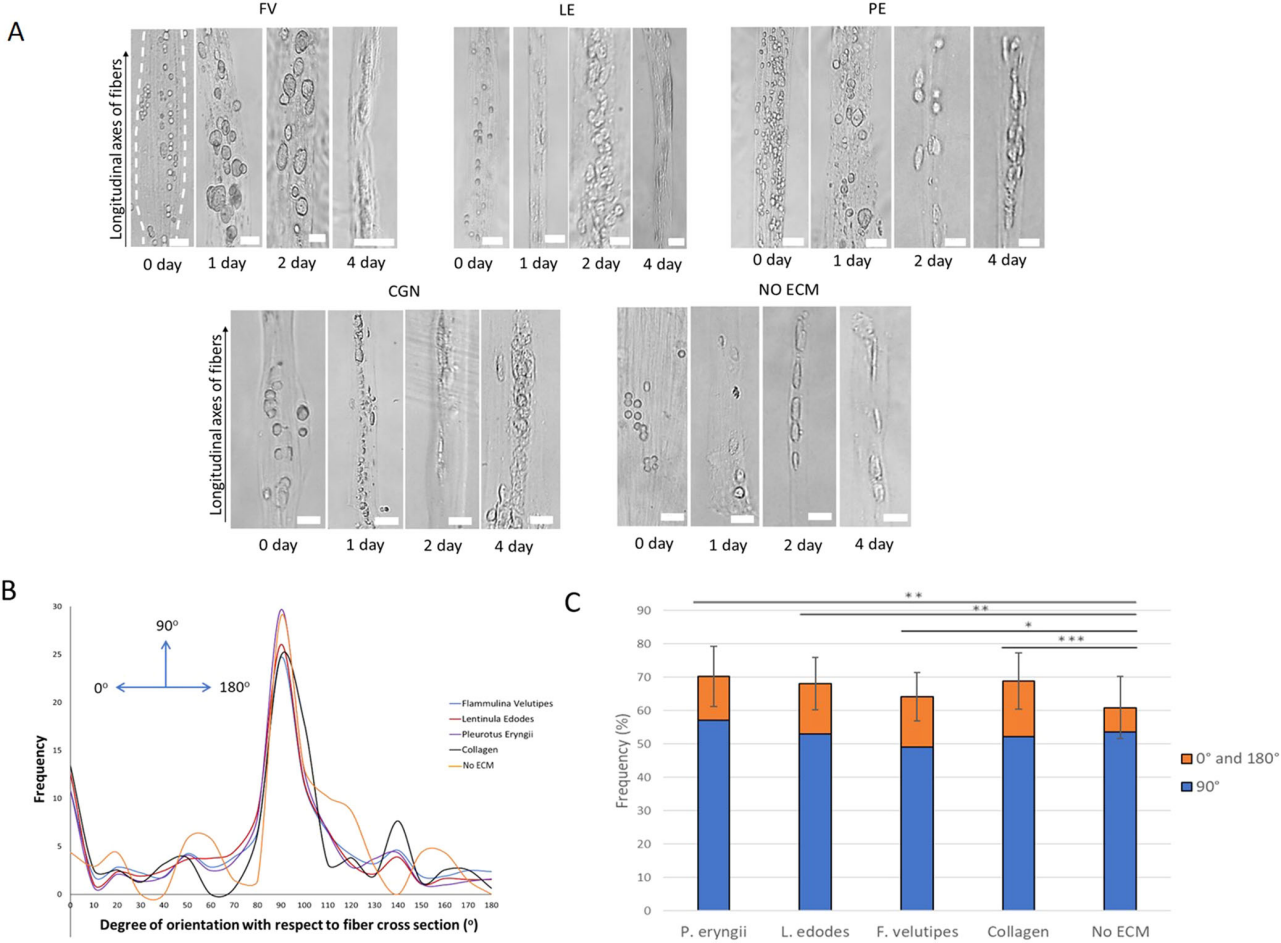
Cell-alignment analysis. **A** Cells encapsulated in chitin-alginate fibers incorporated with fungal particulate extracts (FV *F. velutipes*; LE *L. edodes*; PE *P. eryngii*), fibers with collagen (CGN) and fibers with no ECM. Scale bars represent 100 µm. **B** Plot of cell alignment for the various fiber types (*n* ≥ 12). **C** The frequency of cell alignment with orientation angles of 0° (range 0–10°) and 180° (range 170–180°), and 90° (range 70–110°) (*n* ≥ 3). The error bars represent one standard deviation of uncertainty. Chi-square tests were performed to compare the difference in frequency of alignment between the different sample types. (*P ≤ 0.05; **P ≤ 0.01; ***P ≤ 0.001). **B**, **C** were plotted using cumulative data over all 4 time points (0, 1, 2, 4 days).

Importantly, we had to establish if the cell-adhesion activity present in the crude extracts was retained by the fungus-derived particles (particulate extracts). As these particles are meant to constitute an extracellular matrix (ECM) supporting mammalian cell adhesion, we designed a 3D assay to evaluate cell adhesion, using a fiber cell encapsulation system developed in our laboratory^10^. C2C12 mouse myoblasts were encapsulated in fibers formed by the interfacial polyelectrolyte complexation of water-soluble chitin (WSC) and sodium alginate, incorporating particulate extract isolated from each of the fungal species. These fibers were placed in media for continuous cell culture. Over a period of time, the cells were observed to align themselves along the axes of fibers, in the form of individual cells or aggregates (Fig. 3A). Similar observations have been made for ECM vs non-ECM incorporated fibers encapsulating stem cells in chondrogenic medium^11^. The plots shown in Fig. 3B compare cell orientation for fibers incorporated with protein from different fungi compared to collagen- and non-ECM-incorporated fibers. Two main features could be discerned in these plots:

1. For all types of cells, a large fraction of the cells was aligned parallel to the longitudinal axis of the fibers, resulting in a peak orientation angle of 90°.
2. For fibers containing particulate extract from all three fungal species and fibers with collagen, a higher fraction of the cells/aggregates were oriented at angles of 0° or 180°, compared to “no ECM” fibers. This phenomenon can be explained by a greater degree of cell–matrix compared to cell–cell interactions in fibers containing the extracts, as elaborated in Supplementary Fig. 3–1 and 3–2.

When the proportion of cells/aggregates oriented at angles of 0° and 180°, and 90° were plotted for the different fiber types, higher orientation at 0° and 180° was observed for fibers containing particulate extract from all three fungal species and fibers with collagen than “no ECM” fibers (Fig. 3C). This result suggests that the particulate extracts support cell adhesion, leading to a higher degree of interaction between the cells and the matrix.

To effectively replace animal-derived proteins as a cell-adhesive matrix for mammalian cells, it was important to compare the strength of cell-adhesion afforded by the fungal particulate extract in comparison to animal-derived matrix protein. To do so, *F. velutipes* particulate extract (FVP) and fibronectin were chosen to represent the fungal-derived and animal-derived matrices, respectively.

**Fig. 4:**
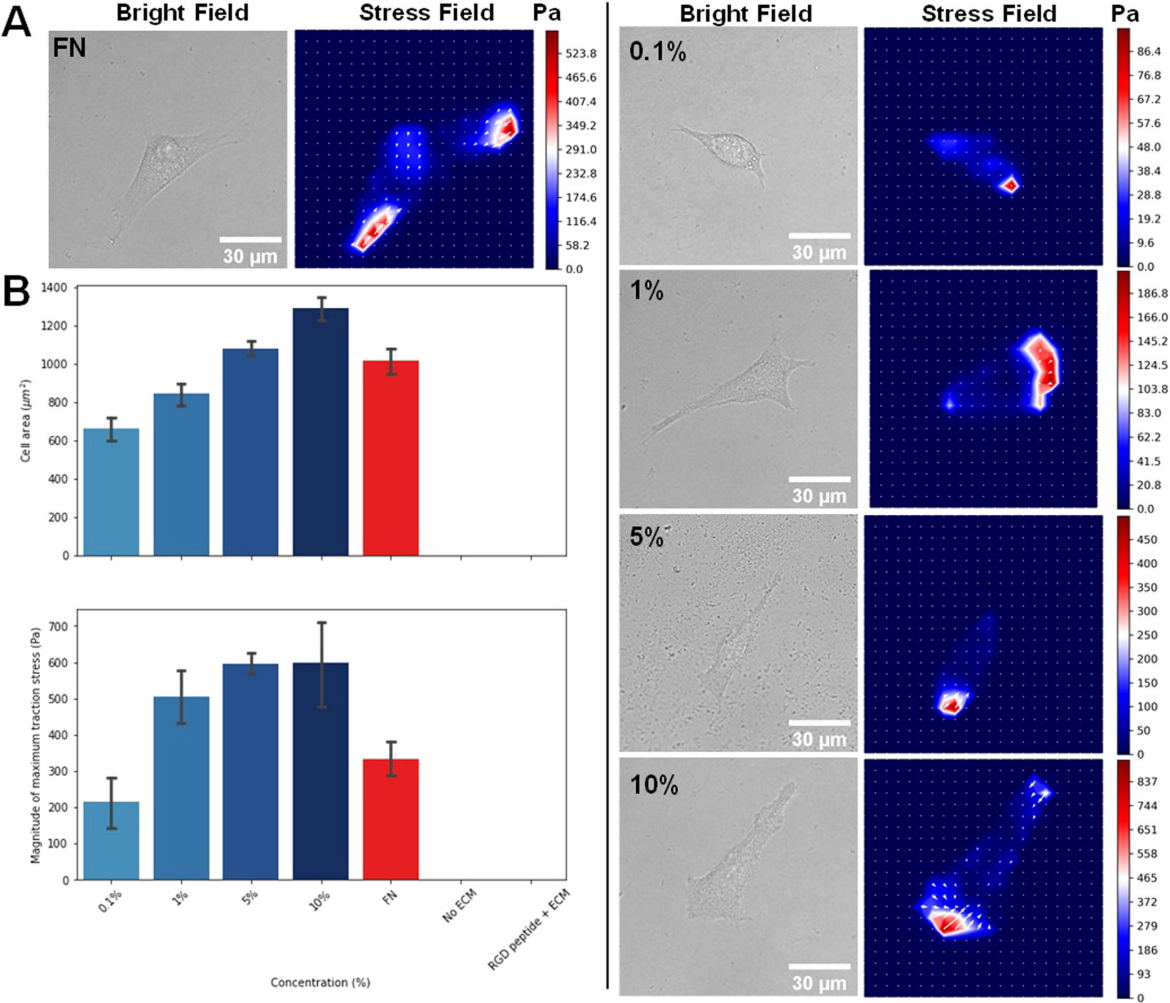
Traction force microscopy. **A** Representative bright-field transmission images (left column) and corresponding traction stress maps (right column) indicating traction stresses for C2C12 cells at 0.1%, 1%, 5 and 10% of FVP (right panel), and with fibronectin (FN) as a positive control (left panel). Stress field maps were obtained from digital image correlation for polyacrylamide hydrogels with Young’s modulus of 14 kPa. Arrows indicate the directions of associated traction stresses. Scale bar represents 30 μm. Color bar indicates the magnitudes of the traction stress in units of Pa. **B** Bar plots for cell area (μm^2^) (top panel) and magnitude of maximum traction stress (Pa) (bottom panel) for C2C12 cells on polyacrylamide substrates coated with 0.1%, 1%, 5 and 10% of FVP or 50 μg/mL fibronectin (FN), or no ECM (sterile water) and RGD peptide with ECM (Fibronectin or FVP) as negative controls (*N* ≥ 14 cells). Error bars represent standard error of the mean.

Traction force microscopy (TFM) was used to measure the traction stresses exerted by C2C12 cells on polyacrylamide hydrogels coated with FVP or fibronectin at varying concentrations while maintaining a constant substrate stiffness. To mimic the physiological stiffness of muscle tissue, we prepared polyacrylamide hydrogel substrates with Young’s modulus of 14 kPa. Cell traction stresses on FVP-coated substrates were comparable to those on fibronectin-coated substrates (Fig. 4). Our maximum traction stress magnitudes corroborate earlier reports on the traction forces of myoblasts using similar substrate stiffness^12^. Consistent with previous studies^13–15^, we found that traction stresses increased proportionally with larger cell areas. C2C12 cells exhibited larger cell areas and exerted higher maximum traction stresses on the gel substrate with increasing concentrations of FVP (Fig. 4B). At the highest FVP concentration tested (10%), the maximum magnitude of traction stress (599 Pa) exerted per C2C12 cell seem to reach a point of saturation whereas at very low FVP concentration (0.1%), the maximum traction stress magnitude merely reached 214 Pa (Fig. 4B). This suggests that the increased density of FVP may promote the generation of traction stress through the enlargement of cellular spread area, recapitulating previous findings with collagen^16^.

As a negative control, we used sterile distilled water (no ECM) on the substrate to provide a baseline quantification. As expected, none of the C2C12 cells adhered to the substrate without ECM, so measurements for cellular traction force were designated to be 0. In addition, we used an integrin-blocking RGD-based peptide, GRGDS, to check for attachment of the cells to the FVP-coated substrate in the presence of the RGD peptide, which inhibits RGD-mediated binding to fibronectin. Consistent with our expectations, there was no cellular attachment to the fibronectin-coated or FVP-coated hydrogel and thus cellular traction force exerted was designated to be 0 for both types of ECM (fibronectin or FVP) in the presence of RGD-based peptide. This confirms the involvement of RGD in FVP, in mediating cellular adhesion to the substrate.

### SDS-PAGE/MS analysis of proteins in the crude and particulate extracts

Having demonstrated cell-adhesive activity of the fungal-derived particulate extracts, we endeavored to characterize their protein composition. As uncovered by our data mining study, the protein particles from fungal species which showed cell-adhesion activity are expected to contain more than one protein type containing the RGD motif (at least six each for *P. eryngii*, *F. velutipes*, and *L. edodes*). While identification of all the proteins to completely elucidate the composition of the different fungal particles would exceed the scope of the present study, we saw the need to characterize the protein content of the extracts as a basis for future investigations and data collection. Towards that end, we carried out peptide fragmentation mass spectrometry coupled with SDS-PAGE, to catalog the likely RGD-containing proteins for the basidiomycete fungal species being investigated.

**Fig. 5:**
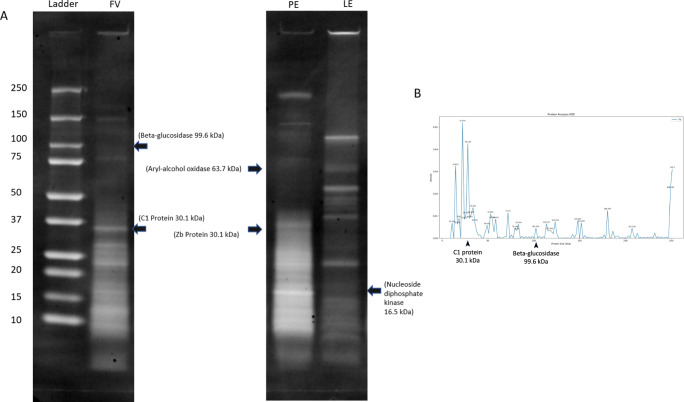
SDS-PAGE analysis of fungal extracts. **A** SDS-PAGE gel showing bands of the protein ladder and crude extracts derived from *F. velutipes* (FV), *P. eryngii* (PE), and *L. edodes* (LE), respectively. RGD proteins of known molecular weight identified by the MS fragmentation experiments were mapped onto the profile. The gels were derived from the same experiment and processed in parallel. **B** Plot of band density vs protein molecular weight (kDa) for crude extract from *F. velutipes*. The putative RGD proteins, C1 protein, and beta-glucosidase have been indicated on this plot.

Briefly, the proteins in the extracts (combined crude and particulate extracts) were enzymatically cleaved and the mass spectra of the resulting peptides and their fragments were measured by mass spectrometry. The list of peptide masses from the spectra was then submitted to Mascot search engine, which compared the measured masses to peptide and their corresponding fragment masses from the theoretical cleavage of proteins in the database. The process yielded a list of possible protein identifications and the confidence of identification (Table 1). In another experiment, the proteins contained in the extracts were also separated by SDS-PAGE, and the major peaks corresponding to RGD proteins of known molecular weight identified by the MS fragmentation experiments, were mapped onto the profile (Fig. 5). For the case of the extract from *F. velutipes*, a plot of band density vs protein molecular weight was obtained from the SDS-PAGE profile and the putative proteins, C1 protein and beta-glucosidase, were mapped onto this plot.

**Table 1:** List of likely proteins present in extracts as predicted by peptide fragmentation analysis.

Organism	Description	Sample type	Accession	Mascot score	Coverage [%]	# Peptides	# Unique peptides	# PSMs
Flammulina velutipes	C1 protein	1	O74163	1157	56	11	11	64
		2	O74163	216	45	8	8	14
		3	O74163	2605	36	9	9	111
		4	O74163	164	37	7	7	12
	Beta-glucosidase	1	G8A544	963	41	27	19	33
		2	G8A544	160	9	7	7	8
		3	G8A544	2317	40	33	19	105
		4	G8A544	187	7	5	5	6
Pleurotus eryngii	C1 protein (Zb protein)	1	Q7ZA62	102	14	3	3	3
		2	Q7ZA62	70	7	1	1	1
		3	Q7ZA62	241	25	5	5	13
	Aryl-alcohol oxidase	1	O94219	129	13	5	5	5
		2	D3YBH4	31	2	1	1	1
		3	O94219	708	14	7	7	19
Lentinus edodes	Nucleoside diphosphate kinase	1	A0A1Q3EJT6	577	61	7	7	17
		2	A0A1Q3EJT6	38	9	1	1	1
		3	A0A1Q3EJT6	1173	54	7	7	41

## Discussion

The broader area of tissue culture, as well as the more specialized field of tissue engineering, have always been supported by animal-derived biomaterials. Culture surfaces and scaffolds to support cell and tissue growth have historically been coated with proteins derived from animals, common examples including collagen (and its denatured form, gelatin), fibronectin, and laminin. Being native components of the ECM, these proteins possess peptide motifs that mediate the adhesion of cells and regulate the subsequent processes of cell proliferation and differentiation^17^. This paper presents fungus-derived cell-adhesive particles (CAFs) as a replacement for animal-derived materials, at least in some applications. These CAFs have the potential to transform the existing tissue culture and tissue engineering industry to a more sustainable one. Importantly, new upcoming industries based on the cultivation of cells to replace the products of animal agriculture require non-animal sources of adhesive factors, and this is where the currently developed CAFs may play a crucial role.

By a simple data mining approach, we discovered that proteins containing RGD are surprisingly ubiquitous throughout the plant and fungi kingdom. Defining an RGD percentage as the percentage of reported RGD-containing proteins for a species over total reported proteins for that same species in the database, close to 90% of all plant and fungi species exhibited an RGD percentage of over 1%. Focusing only on fungal species in this paper, we found the highest percentages of RGD for pathogenic, mycorrhizal and parasitic fungal species. Evidence suggests that the RGD-containing fungal proteins bind to integrin-like receptors on the surface of the plant cell plasma membrane, inducing a decrease in plasma-membrane-cell wall connections and thus reducing the expression of non-specific defense responses during fungal penetration of host cells^18^. This generic function of RGD may explain why an array of fungal proteins with seemingly different functions contain the RGD sequence.

Keeping in mind the food-related applications for the CAFs, we limited our investigations to common and edible fungi with relatively high RGD percentage (>2%), such as *L. edodes* (shiitake), *F. velutipes* (enoki), and *P. eryngii* (king oyster mushroom). We found a simple, inexpensive way to generate particulate extracts comprised mainly of proteins from crude extracts of these fungi, which did not require expensive isolation steps or materials. Increasing the temperature of the crude extracts led to protein denaturation, resulting in the formation of submicron to micron-sized particles that were mostly uniform and negatively charged. These particles are constituted of a mix of RGD-containing as well as non-RGD-containing proteins, which could be treated as the functional and structural components of a fungal ECM, respectively. Denaturation of the proteins would be beneficial for a fungus-derived matrix to be used for mammalian cell culture; while the cell-adhesive function, relying on the primary sequence of the RGD peptide motif would be retained, enzymatic functions of the fungi proteins which rely chiefly on protein 3D conformation would be inactivated. The latter effect minimizes any undesired biological activity of the fungal matrix on mammalian cells. The function of cell adhesion was shown by the alignment of myoblasts in fibers incorporated with the fungal particles, while a quantitative measure of the adhesive strength was obtained by traction stress measurements of cells on CAF-coated polyacrylamide substrates.

From our MS analysis of RGD-containing proteins in the extracts, RGD-containing proteins that were likely to be present were mostly those with cell-adhesive functions (Table 1 and Supplementary Table 1). Amongst these, a protein of ~30 kDa from *F. velutipes* (C1 protein, inositol pyrophosphate synthase) was previously identified to contain the RGD motif from its gene sequence^19^. As many fungal species do not have their complete proteomes mapped compared to model organisms, we relied on TrEMBL databases that contain protein sequences derived computationally from their available genomes or transcriptomes. Consequently, there are proteins that have different names but are identical. For example, C1 protein (UniProt ID: O74163) from *F. velutipes* share 100% sequence identity, isoelectric point, and molecular weight with the Zb protein (UniProt ID: O7ZA62) expressed in *P. eryngii*. We corrected the grouping in Table 1 by choosing one of the protein names as the representative for proteins that share these identities (currently applicable only for C1 and Zb protein). In *P. eryngii*, an aryl-alcohol oxidase with no indicated cell-adhesive function, was also identified. Aryl-alcohol oxidase is an enzyme which both degrades lignin and activates β-glucosidase, potentially exposing the latter’s fibronectin Type III binding sites, which contain RGD^20^. Mapping these identified proteins onto SDS-PAGE, C1 protein from *F. velutipes* appears as a major protein band, supporting its presence in the protein particles and role in mediating the observed cell adhesion (Fig. 5). On the other hand, for the extracts from *P. eryngii* and *L. edodes*, RGD-containing proteins detected in MS do not appear to map to any major band on PAGE. Hence, it could be possible that other peptide motifs apart from RGD are involved in cell adhesion. Homology modeling indicated that the RGD motif was located on the surface for four out of five RGD-containing proteins from Table 1, including C1 protein (Supplementary Fig. 4–1 and 4-2).

Ligation of integrins by the RGD motif and other binding sites in the ECM is crucial to cells, and not just for adhesion. For cells to progress from the G1 to S phase of the cell cycle and thus proliferate, integrin-mediated signaling is required^21^. In growing cells for cultivated meat, a cell culture support material that can bind cellular integrin receptors may be just as important as growth factors, and it is proposed that such supports may even reduce the requirement for the latter. An interesting analogy can be made between the particulate fungal matrices developed in this paper, with the ECM derived from mouse sarcoma (commercially branded as Matrigel/Geltrex) that is widely used for biological studies, and especially 3D cell culture. Both the fungal matrix and Matrigel are composed of more than one protein with cell-adhesive activity. Matrigel is a composite of ECM proteins such as collagen, laminin, fibronectin and entactin. These ECM proteins have other binding sites with specific functions impacting cell activity, however most of them contain RGD sequences for the basic function of mammalian cell adhesion. Unlike the presumably denatured proteins in our fungal protein particles, Matrigel contains proteins which are active, including the ECM proteins and growth factors that may enhance processes of cell growth and differentiation. However, Matrigel is a far less sustainable biomaterial compared to the fungal ECM of this paper. In the production of cell-cultivated food such as cultivated meat, where the underlying paradigm is the production of meat without the breeding and rearing of live animals, an alternative non-animal-derived ECM is desirable.

An inevitable feature of future foods would be the replacement of at least some of the animal protein in our diet with similar protein either from plant-based sources, or via the culture of cells. Various plant-based polymers have been investigated as the base material to construct edible cell culture supports, for example, decellularized scaffolds^22^ and textured soy protein^23^. As these scaffolds per se are unable to support cell adhesion, coating or incorporation with cell-adhesive factors (CAFs) is required. Besides Cardosin A^24^, animal-derived ECM proteins and synthetic or recombinant peptides are the only reported CAFs for this purpose to date^25^. Being readily produced and scaled up, the fungal-derived protein particles presented in this paper are a potential cost-effective alternative for cell-cultivated food applications. This fungal ECM can be derived from edible fungi, or fungi that have been used in other food processes, facilitating their approval in food products. The same CAFs developed in this paper could also find application as more sustainable materials for healthcare. For instance, they offer an insoluble protein form that can be easily incorporated into tissue engineering scaffolds, in a similar way to mammalian ECM.

In summary, we have established a method to screen for, and identify candidate fungal species based on their content of proteins with the cell-adhesive RGD motif. Extraction of proteins from these species followed by a heating step to induce their denaturation led to the formation of protein particles. These exhibited cell-adhesive properties, as demonstrated by the appropriate 2D and 3D cell-adhesion and alignment assays. Such fungus-derived “extracellular matrices” are potentially applicable to diverse areas which require the adhesion of mammalian cells, and are foreseen to benefit the present drive towards sustainability.

## Methods

### RGD data mining

We used RefSeq^1^ (fungi data) and GenBank^2^ (GBPLN, Fungi and Plantae data) for the reasons stated under data source selection. Data was downloaded on 2021/7/15 and 2021/8/16, respectively via FTP; RefSeq: ftp.ncbi.nih.gov/refseq/release/fungi/ (fungi.XX.protein.faa.gz) and GenBank: ftp://ftp.ncbi.nih.gov/ncbi-asn1/protein_fasta (gbplnX.fsa_aa.gz), X representing all applicable numbers. Data was pre-processed by filtering out the redundant proteins in the data source. To simplify our process, we defined redundant protein as the unique protein name in the same organism. Organisms were also removed from our database when their total number of protein sequences was lower than the average number of protein sequences. This process was applied to generate a robust RGD percentage and minimize the scenario when an organism has only one RGD protein but results in a high RGD percentage because the organism has too little protein data. A customized Python script was used to detect the “RGD” motif for every sequence in our database and followed by calculating the RGD percentage for each organism. RGD Percentage is defined as the “Protein RGD Count” divided by the “Protein Count”. “Protein RGD Count” and “Protein count” are defined as the number of proteins which contain RGD in the organism and the number of proteins in the organism, respectively.

### Data source selection

We first used RefSeq as our only data source since it is a non-redundant database and widely used in different studies^3^. However, studies reporting RefSeq are limited to major organisms for which sufficient data is available^4^. For instance, as of April 2019, the RefSeq database only contained 285 fungal genome sequences, although the number of fungal species is estimated to be over 2 million^5^. Till July 2021, only 432 species were found in RefSeq fungal data. Due to the limitation, several common edible fungi which we are interested in (such as *F. velutipes* and *L. edodes*) were not found in RefSeq. As the GenBank protein database contains more species^4^, we included GenBank protein data in our database, enabling us to cover most of the common edible fungi (such as *F. velutipes* and *L. edodes*). However, curation is rarely done for the GenBank database and it contains redundant protein sequences. Thus, by including RefSeq and GenBank in our database, followed by filtering out the sequences with the same protein name within the same species, both databases complemented each other and provided informative results.

### Isolation of CAFs from fungi

The fungal mushrooms (Flammulina velutipes, Grifola Frondosa, Lentinus edodes, Pleurotus eryngii, Pleurotus Ostreatus) were purchased from local supermarkets (Cold storage, NTUC Fairprice). Fungi were frozen by placing them in liquid nitrogen and subsequently blended into powder using a blender. In total, 14 g of powdered fungi was added to 25 ml of deionized water in a 50 ml Falcon® Conical Centrifuge Tube and shaken to form a homogenous suspension. The suspension was then placed into a box of ice and subject to direct sonication for 30 min (Vibracell ultrasonic processor), at 10% amplitude to disrupt the fungal cell walls and release protein into the solution (crude extract). After the sonication process, the suspension was centrifuged at 10,000 rpm, 4 °C for 10 min. The water-insoluble fungal biomass was separated as a pellet at the bottom of the falcon tube. The supernatant was decanted into a 100 ml blue-cap bottle and subject to heating on a hotplate at 200 °C for 20 min. Upon heating, a white precipitate was formed. The solution was then removed from the hotplate and cooled down to room temperature before centrifuging for 10 min at 10,000 rpm. After removing the supernatant, 10 ml of deionized water was added to the pellet and vortexed to obtain a homogeneous dispersion. The suspension was then subject to another round of centrifugation to wash the microparticles and the remaining pellet was collected as the cell-adhesive factors (CAFs) (particulate extract).

### Dynamic light scattering (DLS) analysis

The total crude extract was prepared using 3.5, 7, and 14 g fungal powder with our protocol (without heating) in 4 °C Tris-MgCl_2_ buffer (0.01 M Tris, 5 mM MgCl_2_). The total crude extract (1 ml) was loaded into a DLS reusable cuvette. Z-average of the total crude extract was measured using a Malvern Zetasizer Nano Z690 at 60, 70, 80, and 90 °C. In total, 36 rounds of DLS experiments was conducted for each organism, i.e., a triplicate of 12 combinations of different crude extract concentrations and heating temperatures. The development of the protein aggregation model is described in Supplementary Information 3.

### Cell encapsulation in polyelectrolyte complex fibers

Sodium alginate (medium molecular weight, Sigma), water-soluble chitin (50% acetylation from Chitin of fungal origin, Glentham, UK). Both alginate and water-soluble chitin solutions were prepared in deionized water and autoclaved for 2 h before usage. The murine skeletal muscle cell line C2C12 was purchased from American Type Cell Culture (Manassas, VA). C2C12 myoblast cultures were maintained for 2–3 weeks in DMEM (Gibco®) supplemented with 10% fetal bovine serum (FBS, GEMINI) and 1% penicillin-streptomycin-fungizone (antibiotic-antimycotic Gibco®). Prior to starting the experiment, C2C12 cultures were maintained in T75 flasks (Corning) inside an incubator at 37 °C in a humidified atmosphere containing 5% CO_2_. Cells were dissociated from the culture flasks at 50–75% confluence with 0.25% trypsin (Gibco®), resuspended in DMEM containing 10% serum, seeded in new T75 flasks, and the medium was changed every 2 days.

C2C12 cells were encapsulated and cultured within water-soluble chitin-sodium alginate polyelectrolyte complex fibers incorporating CAFs, as described in the following process. The nitrocellulose paper used in this experiment was soaked in 70% ethanol overnight, then sterilized by UV irradiation for 1 h. A 15 μL droplet of 2% CAFs incorporated in 1% medium molecular weight alginate was dispensed using a 200 μL pipette onto a parafilm surface, then a droplet of 5 μL of cell suspension (1.0 × 10^6^ cells/mL) was introduced into the same bead. Another droplet of 20 μL of water-soluble chitin was placed adjacent to the initial bead, and a pair of forceps was used to gently bring the two polyelectrolyte droplets into contact to form an interface, before drawing a fiber strand upwards. The fiber strand was then wrapped around a rectangular frame constructed from nitrocellulose paper, before placing into a six-well plate containing 2 mL of media per well. The alginate-water-soluble chitin polyelectrolyte complex fibers were examined under the light microscope to confirm the encapsulation of C2C12 cells, and incubation was carried out at 37 °C for 6 days. Micrographs were obtained periodically and ImageJ software was used to convert the micrographs to black-and-white images. The latter were digitized and input to a Matlab program that was used to generate cell-alignment plots. For the cell-alignment plot of Fig. 3B, we aggregated the results for each sample type over all time points (*n* ≥ 12).

### Cell traction stress analysis

Polyacrylamide hydrogels containing 6-aminocaproic acid (ACA) (Sigma-Aldrich, #A2504) with Young’s modulus of 14 kPa were fabricated based on the protocol described in ref. ^26^. In addition, red fluorescent microbeads of 0.2 μm diameter (Invitrogen, #F8810) were added to the gel mixture to allow the visualization of substrate deformation and quantification of traction stresses exerted by the cell. 6.25 μL of gel solution was pipetted onto silanized circular coverslips (25 mm diameter) and left to polymerize after being covered with a non-silanized circular coverslip (15 mm diameter) each. The subsequent procedure for hydrogel activation follows the steps outlined in ref. ^26^.

To measure traction stress magnitudes exerted by C2C12 cells on protein-coated hydrogel substrates, traction force microscopy (TFM) was conducted. After functionalization of the UV-sterilized hydrogels (∼50 μm thick) with 50 μg/mL fibronectin or FVP diluted to 0.1%, 1%, 5 and 10% in sterile-filtered PBS (Gibco), the gels were submerged in 0.5 M ethanolamine (Sigma-Aldrich) for 30 min at 4 °C. Hydrogels were then rinsed twice with HEPES buffer (0.5 M HEPES, pH 9.0) and thrice with PBS in a sterile hood. C2C12 cells were seeded onto the protein-coated hydrogels for 24 h at a density of 50,000 cells/coverslip. For the negative control, C2C12 cells were incubated at 37 °C and 5% CO_2_ with the RGD-based peptide, GRGDS (#4189, Peptide Institute) at 2 mg/mL for 90 min as stated in (Oharazawa et al., 2005) before being seeded onto the protein-coated hydrogels. Coverslips with cells adhered to the hydrogel were subsequently mounted in a perfusion chamber (Chamlide, #CM-B25-1PB). Live-cell image acquisition was done using the Leica Stellaris 8 confocal microscope with a ×63 oil-immersion objective (Numerical Aperture 1.4) (Leica) and images were processed with Fiji software package (ImageJ). A detailed description of the 2D traction stress calculations had been published previously^26^. Briefly, we acquired two sets of images of the embedded fluorescent microbeads on the gel surface before and after cell detachment with TrypLE Express (Gibco). Digital image correlation algorithm^27,28^ was applied to determine the 2D displacement vectors, as a result of gel deformations via cellular traction stresses. Bead displacements in the *x–y* plane were then superimposed over the projected cell area. Upon computing the displacement field, the traction stress field was estimated as an analytical solution to the inverse Boussinesq problem^29,30^.

### Proteomics sample preparation

*F. velutipes*, *L. edodes*, and *P. eryngii* samples were prepared for proteomics analysis as described in the above section “Isolation of CAFs from fungi’”. For whole-body samples, samples were resuspended in 50 mM HEPES, pH 8.0 containing 8 M urea and homogenized using a mechanical homogenizer on ice. Samples were further lysed using a probe sonicator (Vibracell, Sonics and Materials, Inc.) on ice at 22% amplitude for 2 min using 5 s pulse and 5 s pause cycles. Protein concentration from lysis of whole-body samples were measured using bichinchoninic acid (BCA) assay. An aliquot (~100 µg) of lysates was taken for reduction and alkylation by the addition of 10 mM final concentration of tris(2-carboxyethyl)phosphine (TCEP) and 55 mM final concentration of 2-chloroacetamide (CAA) before incubation in the dark for 30 min. Samples were diluted eightfold by the addition of triethylammonium bicarbonate (TEAB), pH 8.5 to reduce the concentration of urea for protein digestion. Samples were then added with endoproteinase LysC (2 µg final amount) and incubated at 37 °C for 3 h followed by trypsin (2 µg final amount) for 37 °C overnight. Digestion was terminated by adding 1% (v/v) final concentration of trifluoroacetic acid (TFA) to the samples, followed by desalting using Oasis HLB cartridge (1 cc/30 mg sorbent, Waters Corporation). The cartridge was equilibrated with 100% (v/v) acetonitrile and washed with 0.1% (v/v) formic acid in water before loading the peptides. Bound peptides were further washed with 0.1% (v/v) formic acid in water and eluted with 0.1% formic acid in 60% (v/v) acetonitrile. Eluted peptides were dried by centrifugal evaporation for MS analysis.

For SDS-PAGE samples, each sample lane was cut using a clean scalpel into pieces based on the following molecular weight ladder sizes: >100, 50–100, 30–50, 20–30, and <20 kDa. Each piece was further cut into smaller pieces of ~1 mm^3^ and transferred into clean microfuge tubes. Gel pieces were destained using 50% (v/v) ethanol in 100 mM TEAB, pH 8.5 with gentle agitation at 25 °C for 5 min thrice and further destained using 100% (v/v) ethanol twice. At the end of the destaining step, ethanol was removed and the gel pieces were rehydrated using 20 mM TCEP in 100 mM TEAB, pH 8.5 and incubated on a heating block at 55 °C for 20 min to reduce disulfide bridges. Samples were cooled to 25 °C before the addition of a final concentration of 55 mM CAA and incubated in the dark for 30 min. After reduction and alkylation, the supernatant was removed and the samples were washed with 100 mM TEAB, pH 8.5 twice and finally added with a sufficient volume of 100 mM TEAB, pH 8.5 containing a final concentration of 12.5 ng/µl trypsin for digestion at 37 °C overnight. Samples were acidified by addition of a final concentration of 1% (v/v) TFA and the supernatant were transferred to new clean tubes. Peptides were then extracted from the gel pieces by addition of 30% acetonitrile in 3% TFA with agitation for 10 min. This was repeated twice and the supernatants were transferred to the same tubes as before each time. Peptides were further extracted by the addition of 100% acetonitrile with agitation for 10 min. This was also repeated twice, and the supernatants were transferred to the same tubes as before each time. Samples were dried using vacuum centrifugation for MS analysis.

### Tandem mass spectrometry analysis

Dried samples were resuspended in 10 µl of 2% (v/v) acetonitrile containing 0.06% (v/v) trifluoroacetic acid and 0.5% (v/v) acetic acid and transferred to an autosampler plate. Online chromatography was performed in an EASY-nLC 1000 (Thermo Fisher Scientific) liquid chromatography system using single-column setup and 0.1% formic acid in water and 0.1% formic acid in 99% acetonitrile as mobile phases. Fractions were injected and separated on a reversed-phase C18 analytical column (Easy-Spray, 75 µm inner diameter × 50 cm length, 2 µm particle size, Thermo Fisher Scientific) maintained at 50 °C and using a 2–27% (v/v) acetonitrile gradient over 45 min, followed by an increase to 55% over the next 15 min, and to 95% over 5 min. The final mixture was maintained on the column for 5 min to elute all remaining peptides. The total run duration for each sample was 70 min at a constant flow rate of 300 nl/min.

Data for whole-body samples were acquired using an Orbitrap Fusion mass spectrometer (Thermo Fisher Scientific) using data-dependent mode. Samples were ionized using 2.1 kV and 300 °C at the nanospray source. Positively-charged precursor signals (MS1) were detected using an Orbitrap analyzer set to 60,000 resolution, automatic gain control (AGC) target of 400,000 ions, and maximum injection time (IT) of 100 ms. Precursors with charges 2–7 and having the highest ion counts in each MS1 scan were further fragmented using collision-induced dissociation (CID) at 35% normalized collision energy. Fragment signals (MS2) were analyzed by the ion trap analyzer at an AGC of 15,000 and maximum IT of 50 ms. Precursors used for MS2 scans were excluded for 90 s to avoid re-sampling of high-abundance peptides. The MS1–MS2 cycles were repeated every 3 s until completion of the run.

For SDS-PAGE-extracted samples, they were acquired using a Q Exactive HFX mass spectrometer (Thermo Fisher Scientific) using data-dependent mode. Parameters were identical to the acquisition on Orbitrap Fusion Lumos with the following changes: precursor signals (MS1) were detected using an Orbitrap analyzer set to 60,000 resolution, automatic gain control (AGC) target of 3,000,000 ions, and maximum injection time (IT) of 50 ms. Precursors with charges 2–5 and having the highest ion counts in each MS1 scan were further fragmented using higher-energy collision dissociation (HCD) at 28% normalized collision energy. Fragment signals (MS2) were analyzed by Orbitrap analyzer at a resolution of 7500, AGC of 100,000, and maximum IT of 15 ms. Precursors used for MS2 scans were excluded for 30 s to avoid re-sampling of high-abundance peptides. The MS1–MS2 cycles were repeated every 40 MS2 scans until completion of the run.

### Proteomics data analysis

Proteins were identified using Proteome Discoverer™ (v2.4, Thermo Fisher Scientific). Raw mass spectra were searched against *F. velutipes*, *L. edodes*, and *P. eryngii* primary protein sequences retrieved from UniProt (June 11, 2021). As these organisms were not widely studied, the TrEMBL databases (computationally imputed sequences) were used instead of Swiss-Prot databases (manually curated sequences) due to a lack of experimentally-validated protein sequences. Carbamidomethylation on Cys was set as a fixed modification; deamidation of Asn and Gln, acetylation on protein N termini, and Met oxidation were set as dynamic modifications for the search. Trypsin/P was set as the digestion enzyme and was allowed up to three missed cleavage sites. Precursors and fragments were accepted if they had a mass error within 10 ppm and 0.8 Da, respectively. Peptides were matched to spectra at a false discovery rate (FDR) of 1% (strict) and 5% (relaxed) against the decoy database. The search result was exported and further processed with the RGD data mining protocol (with UniProt database), as mentioned in the earlier section, to identify mass spectrometry-detected proteins which contain the RGD motif. The molecular weights (MW) and pI values of the proteins containing RGD motifs were also extracted from the database.

### Reporting summary

Further information on research design is available in the Nature Research Reporting Summary linked to this article.

## Supplementary information

Supplementary Information

Reporting Summary

## Data Availability

The authors can confirm that all relevant data are included in the paper and/or its Supplementary Information.

## Code availability

The codes that support the findings in this paper are available in the Supplementary Information (for image processing) or from the corresponding author upon reasonable request (for data mining).
