# Supplementary material for: Fungus-derived protein particles as cell-adhesive matrices for cell-cultivated food
Source: NPJ Sci Food. 2023 Jul 13;7:34. doi: 10.1038/s41538-023-00209-y (PMC10344888; doi:10.1038/s41538-023-00209-y)
Supplement: Supplementary file 1 — Supplementary Information [file 41538_2023_209_MOESM1_ESM.docx]

**Supplementary Information**

**Fungus-derived protein particles as cell-adhesive matrices for cell-cultivated food**

Yu Xing Teo^1,#^, Kah Yin Lee^1,#^, Corinna Jie Hui Goh^2^, Loo Chien Wang^3^, Radoslaw M. Sobota^3^, Keng-Hwee Chiam^2^, Chan Du^1^*, Andrew C. A. Wan^1^*


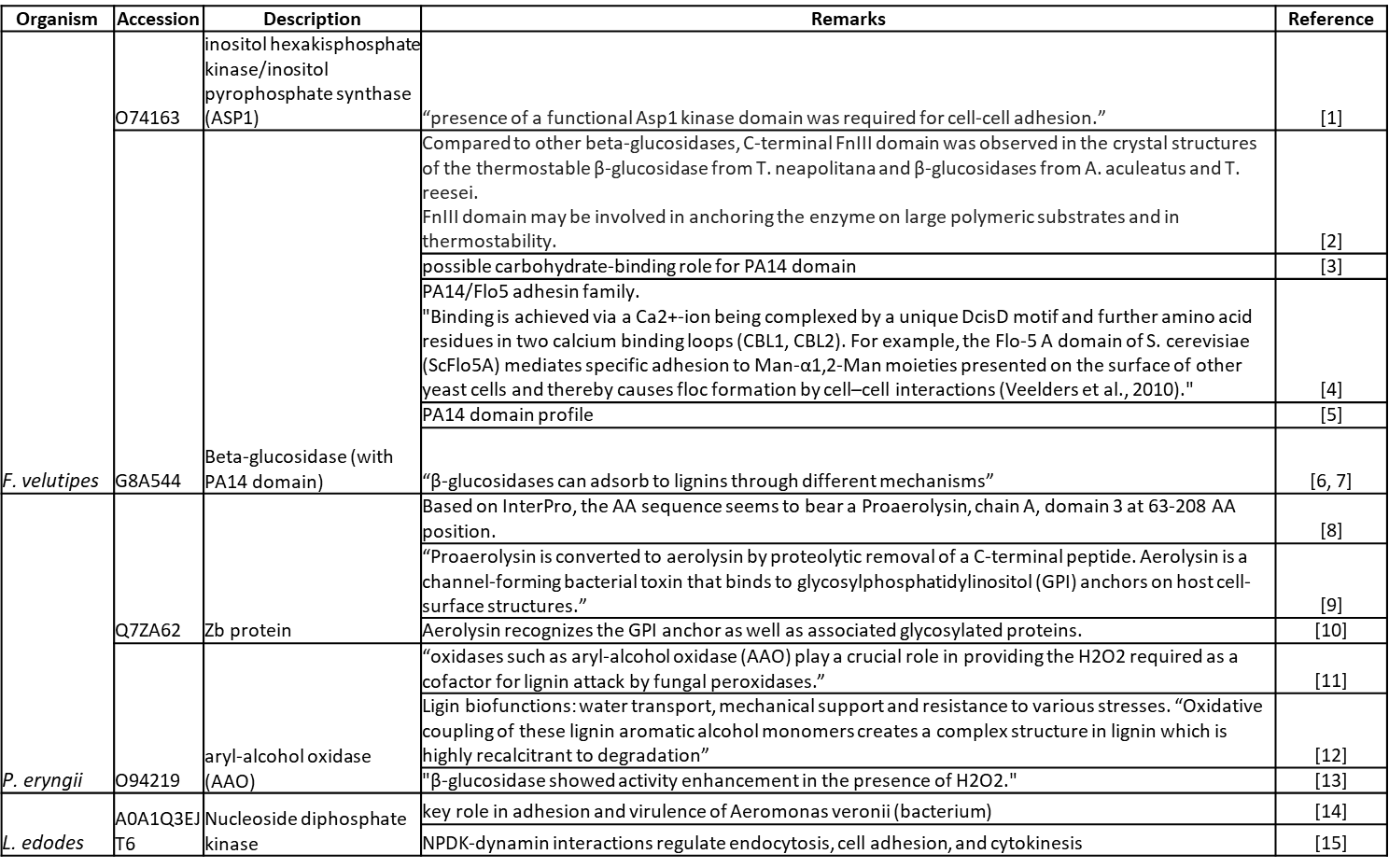


Supplementary Table 1: Reported function of likely proteins present in extract/particles, as identified by MS analysis

1. **Cell Adhesion on Plates Coated with Crude Fungal Extracts**

Primary human skeletal muscle cells (SMCs) and human breast carcinoma cell line, MCF7 were obtained from ATCC. Cells were seeded onto NUNC Maxisorp polystyrene microtiter plate surfaces at a cell density of 5 x 10^4^ cells per well.

**
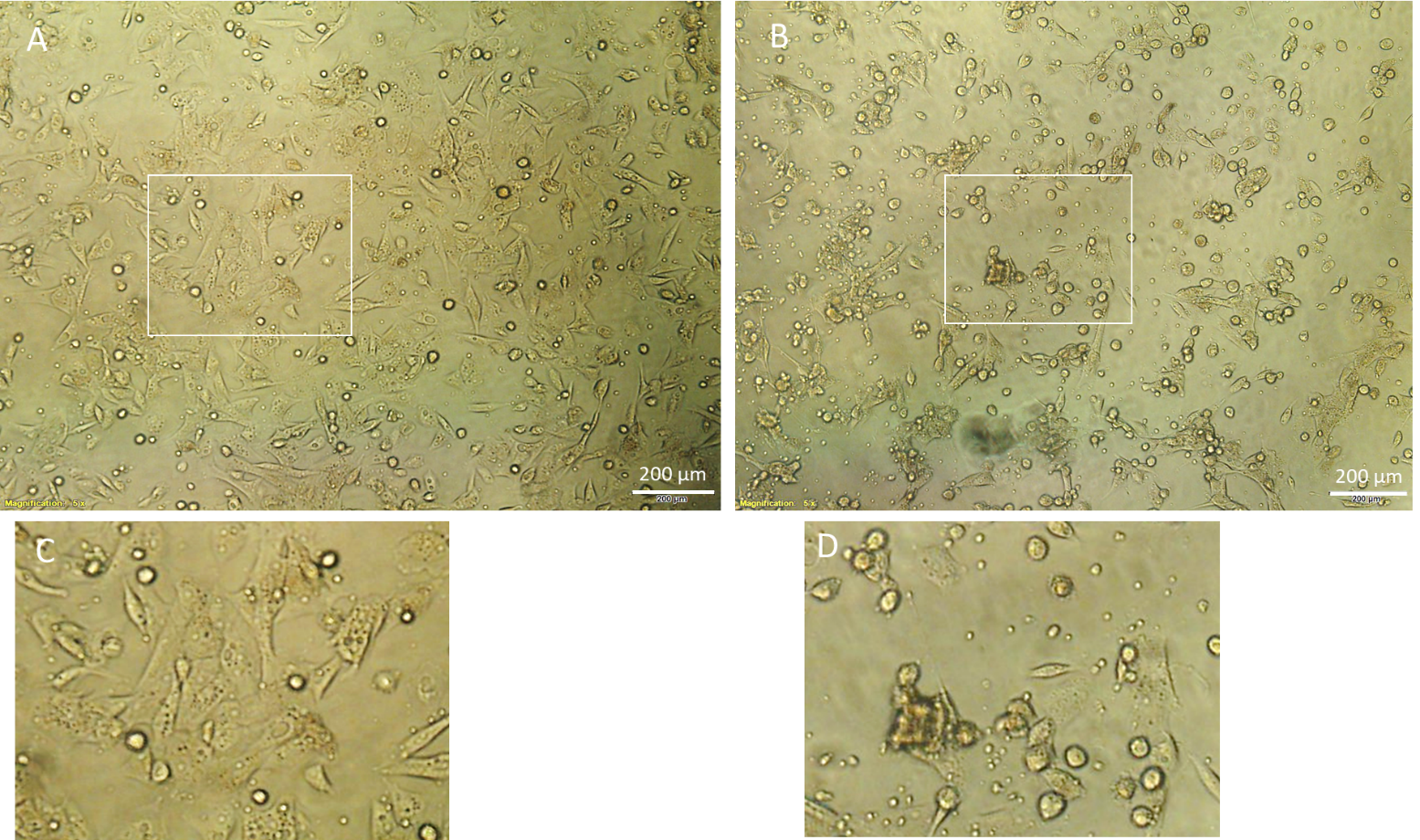
**

Supplementary Figure 1-1. Skeletal muscle cells on (A) surface coated with FV crude extract , (B) non-coated surface, after 3 h of cell culture. (C) Inset of (A) at higher magnification, showing a large proportion of adhered, spread-out cells; (D) Inset of (B) at higher magnification, where most of the cells remain rounded.


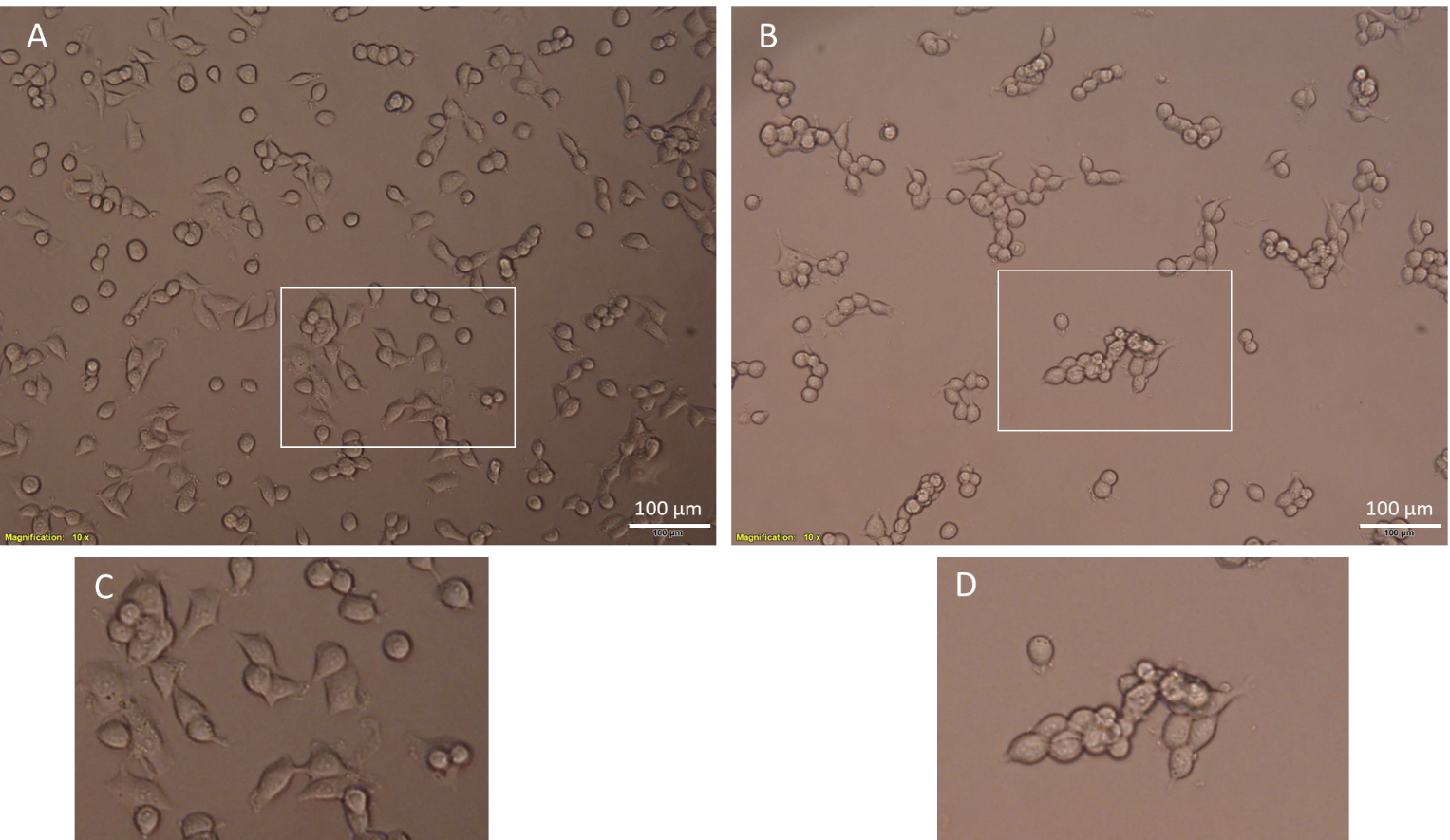


Supplementary Figure 1-2. MCF7 cells on (A) surface coated with FV crude extract, (B) non-coated surface, after 3 h of cell culture. (C) Inset of (A) at higher magnification, with a denser population of cells owning an adherent, spread out morphology ; (D) Inset of (B) at higher magnification, where less cells are present, with a rounded morphology and tendency to form clusters.


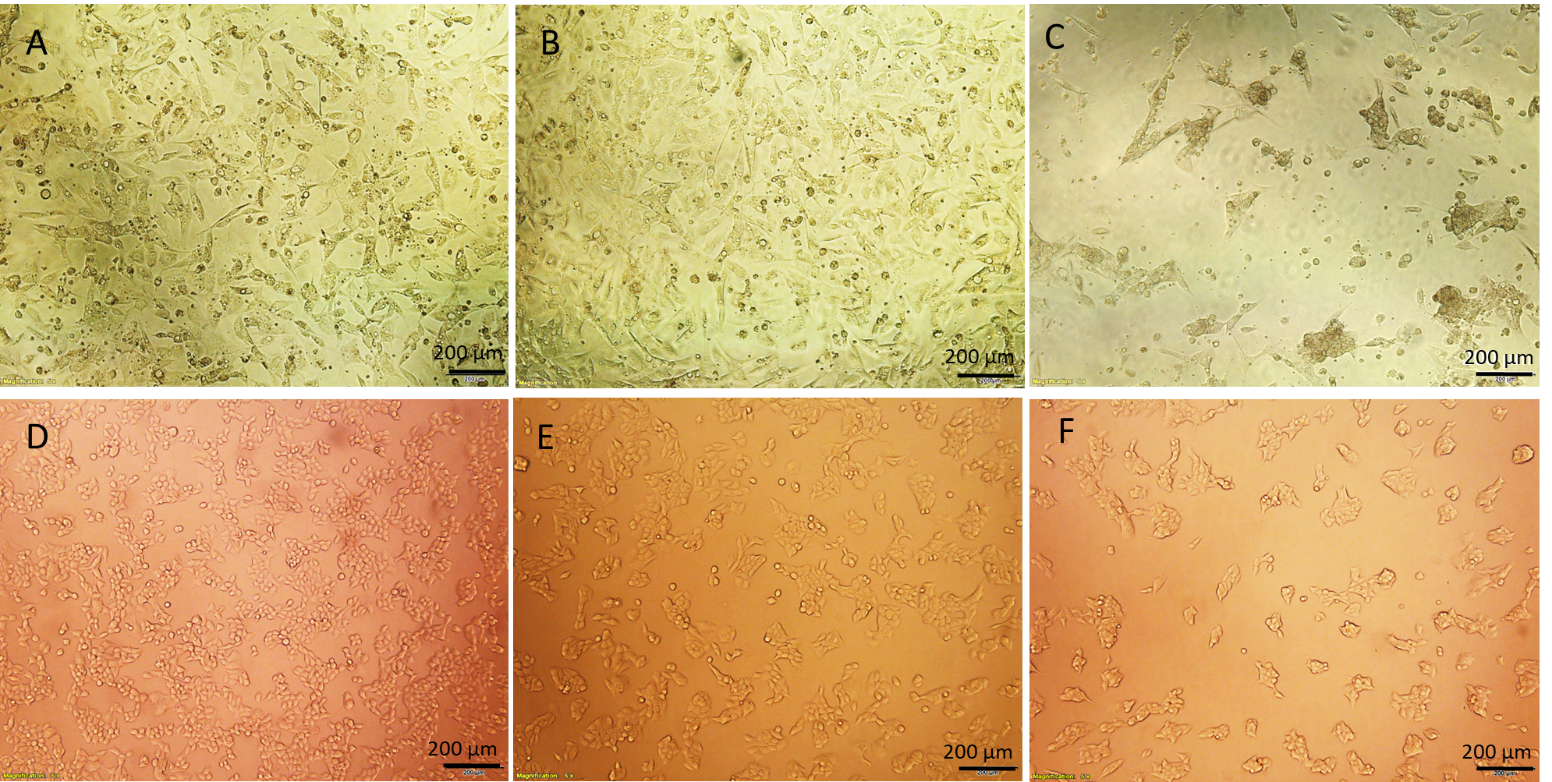


Supplementary Figure 1-3. Skeletal muscle cells on surface coated with (A) FV crude extract , (B) LE crude extract, (C) non-coated surface, after 18 h of cell culture. MCF7 cells on surface coated with (D) FV crude extract, (E) LE crude extract, (F) non-coated surface, after 18 h of cell culture.

**2. Modelling of Protein Aggregation Dynamics of Fungal Extracts Under Different Conditions**

Fungal powder was obtained by freezing fungal biomass using liquid nitrogen, followed grinding with a blender. 2 g of powder from each fungal species was mixed with 1 mL of chilled (4^o^ C) Tris-EDTA in a 5ml tube. The suspension was sonicated on ice for 50mins, then centrifuged at 15000rpm (4^o^ C) for 10mins. The supernatant was transferred into a 2ml tube and kept on ice. The protein concentration was measured using BCA Protein Assay Kit according to manufacturer’s instructions. The results (shown below) suggest that protein aggregation was more a function of the protein types present in the supernatants of each fungal species rather than the concentration of the proteins.  L. Edodes, with the highest crude extract protein concentration, precipitated at the slowest rate, whereas P. eryngii, with the lowest crude extract protein concentration, precipitated at the fastest rate.

| Fungus | Mean (ug/ul) | Standard Deviation |
| --- | --- | --- |
| L. edodes | 29.18444 | 6.202456 |
| P. eryngii | 12.04267 | 4.166173 |
| F. velutipes | 19.64148 | 8.58265 |
| BSA control (1µg/ul) | 0.920725 | 0.238244 |

Concentration of protein in crude extract of fungi via BCA kit

| Treatment vs. Control | P-Value | Statistical Significance of t-test |
| --- | --- | --- |
| L. edodes (Treatment) vs. P. eryngii (Control) | 0.005231 | ** |
| L. edodes (Treatment) vs. F. velutipes (Control) | 0.126392 | ns |
| L. edodes (Treatment) vs. BSA control (1µg/ul) (Control) | 0.002765 | ** |
| P. eryngii (Treatment) vs. F. velutipes (Control) | 0.180822 | ns |
| P. eryngii (Treatment) vs. BSA control (1µg/ul) (Control) | 0.041797 | ** |
| F. velutipes (Treatment) vs. BSA control (1µg/ul) (Control) | 0.02222 | * |

P-value of the two-tail unequal variance t-test of the BCA result, showing that *L. edodes* (LE) had a significantly higher protein concentration than *P. eryngii* (PE). *: P ≤ 0.05; **: P ≤ 0.01; ns: P > 0.05.


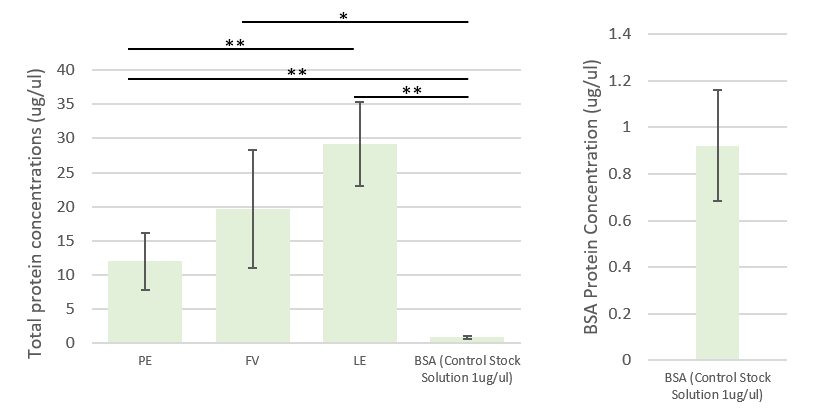


Supplementary Figure 2-1. Total protein concentration from different fungus extract. 2g fungi was dissolved in 1ml chilled (4^o^ C) Tris-EDTA for adhesion factor isolation. Protein concentrations of the different fungi were measured using the BCA kit, and two tail t-tests with unequal variance were performed. The error bars represent one standard deviation of uncertainty. The error bars represent one standard deviation of uncertainty. The P values are represented by asterisks (*); *: P ≤ 0.05; **: P ≤ 0.01. The total protein concentration of each fungus is the mean of at least three independent experiments (N ≥ 3).

*Flammulina velutipes*

**Comparison of Various Fungal Biomass Concentrations**


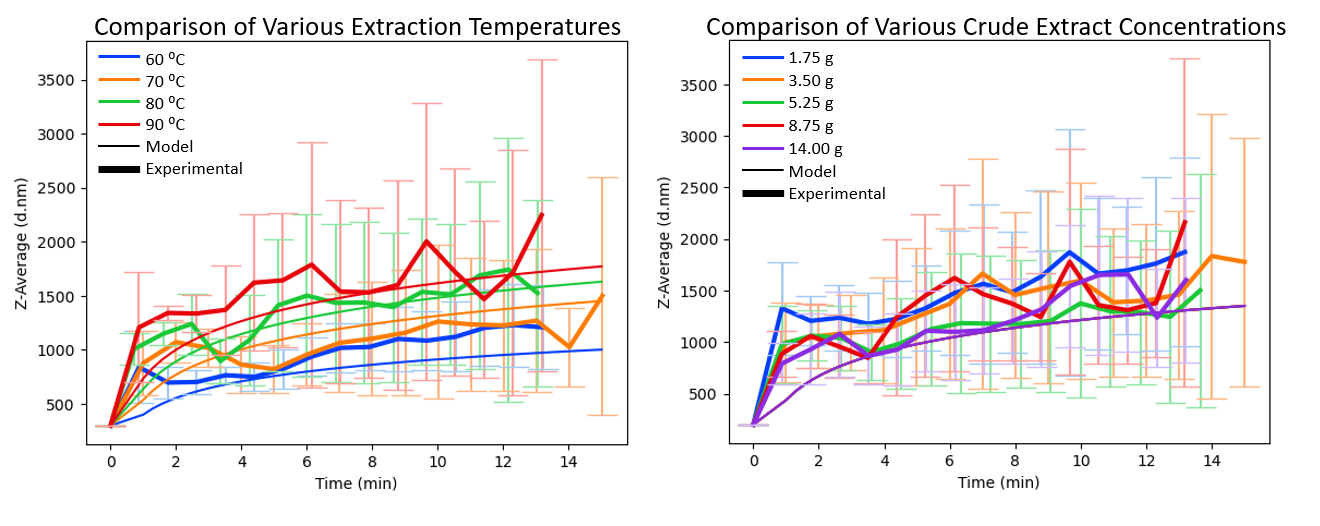


*Lentinus edodes*

**Comparison of Various Fungal Biomass Concentrations**


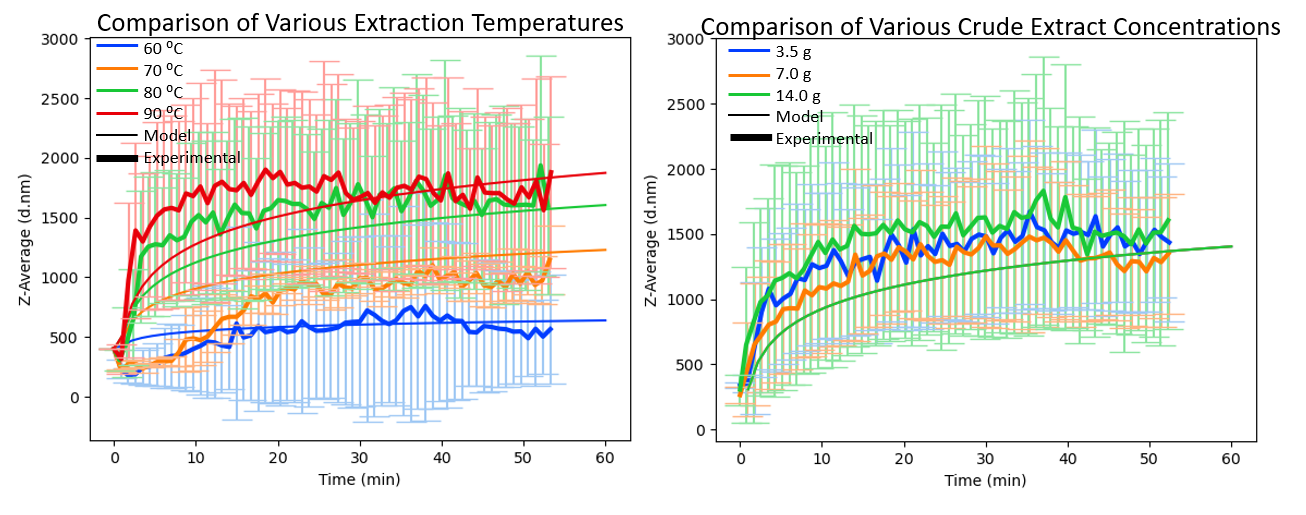


*Pleurotus eryngii*


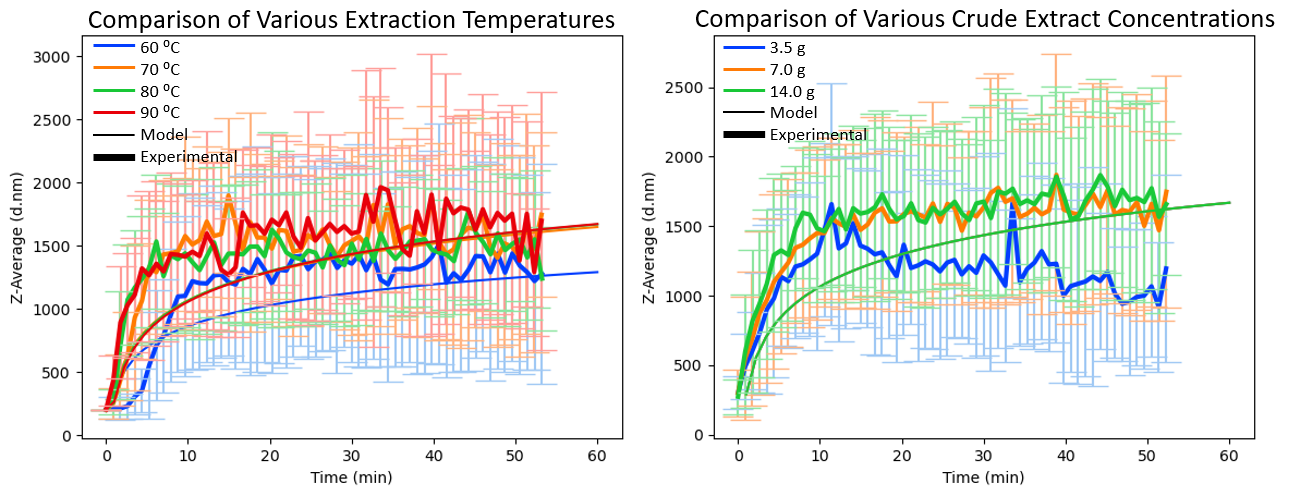


**Comparison of Various Fungal Biomass Concentrations**

Supplementary Figure 2-2: Model prediction and validation of protein aggregation dynamics for three basidiomycete species under different conditions of temperature and protein extract concentrations. The aggregation dynamics of each fungus was analysed by at least three independent experiments (N ≥ 3). The error bars represent one standard deviation of uncertainty.

| Treatment vs. Control | P-Value | Statistical Significant of T-test |
| --- | --- | --- |
| 60 ⁰C (Treatment) vs. 70 ⁰C (Control) | 0.855034 | ns |
| 60 ⁰C (Treatment) vs. 80 ⁰C (Control) | 0.005845 | ** |
| 60 ⁰C (Treatment) vs. 90 ⁰C (Control) | 0.024394 | * |
| 70 ⁰C (Treatment) vs. 80 ⁰C (Control) | 0.002678 | ** |
| 70 ⁰C (Treatment) vs. 90 ⁰C (Control) | 0.014515 | * |
| 80 ⁰C (Treatment) vs. 70 ⁰C (Control) | 0.834117 | ns |

P-value table for different aggregation temperatures in Lentinus edodes. The P values are represented by asterisks (*); *: P ≤ 0.05; **: P ≤ 0.01; ns: P > 0.05.

**Comparison of Various Fungal Biomass Concentrations**


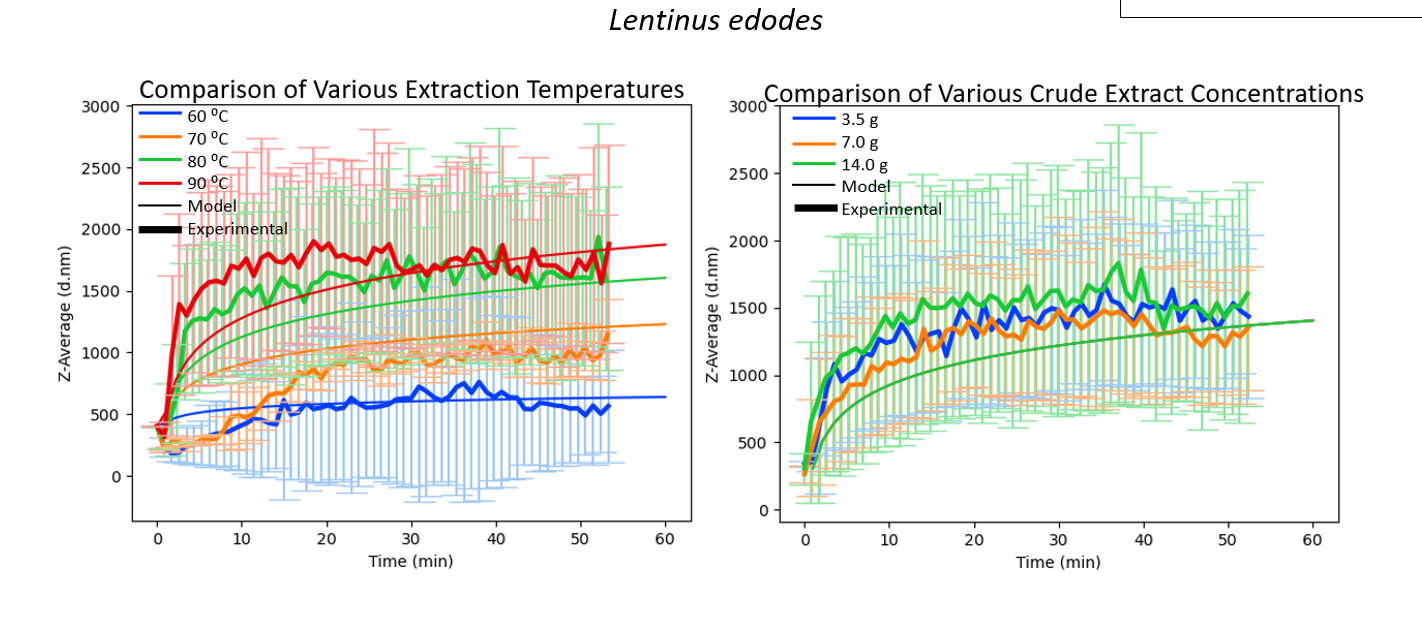


Supplementary Figure 2-3: ANCOVA tests were performed to compare the aggregation dynamics for L. edodes at different temperatures, within the initial 10mins. The statistical test results suggested that particle sizes at 90^o^ were significantly different compared to those at 60^o^ and 70^o^, with P-value < 0.05. Particle sizes at 80^o^ were also significantly different compared to those at 60^o^ and 70^o^, with P-value < 0.01. Statistical treatment of results of protein aggregation experiment for L. edodes showing that aggregation increased at higher temperatures. There was no clear dependence of aggregation on fungal-biomass concentration. The aggregation dynamics of each fungus was analysed by at least three independent experiments. The error bars represent one standard deviation of uncertainty.

| ANCOVA Test | P-value |
| --- | --- |
| L. edodes (various concentrations) | 0.8491 |
| P. eryngii (various concentrations) | 0.9057 |
| F. velutipes (various concentrations) | 0.9809 |

P-value table for the aggregation dynamic of different concentrations for each fungi were analyzed by ANCOVA test. Table shows that there is no significant concentration-dependence.

*Protein Aggregation Model*

Supervised machine learning is used to fit the Z-averages of each organism obtained from Zetasizer measurements with the “curve_fit” function provided by Scipy library^16^. An exponential curve (Eq. 1) is used in the “curve_fit” function.

$Z=p_{1}e^{p_{2}t}$ (1)

Where *t* is the measuring time and *Z* is the Z-average. While $p_{1}$ and $p_{2}$ are the parameters associated with heating temperature and concentration. Then, a logarithmic model (Eq. 2 and Eq. 3) is used to fit the $p_{1}$ and $p_{2}$parameters,

$p_{1}=a\log_{e} \left( b\left( T-T_{0} \right) \right)+c$ (2)

$p_{2}=d\log_{e} \left( e\left( C-C_{0} \right) \right)+f$ (3)

Based on preliminary results (data not shown), $p_{1}$ is depend on the temperature (*T*) and initial concentration ($T_{0}$), while $p_{2}$ is depend on the crude extract concentration (*C*) and crude extract initial concentration ($C_{0}$). While $a$ to $f$ are the parameters that need to be fitted. After fitting parameters $a$ to $f$, the Z-average at particular $T_{0}$, $T$, $C_{0}$, $C$ and particular time is predicted by Eq. 1-3.

**3. Cell Alignment Assay**

The C2C12 myoblast cell images of the experiment were taken using cellSens imaging software with Olympus Microscope DP70 camera, Model IX71. The microscopic images of the cell were first transformed to binary image form before the orientation information of the cell were obtained, to quantify cell alignment. The orientation distribution of the myoblast cells is then calculated by using custom-made functions in MATLAB® Online™ (https://www.mathworks.com/products/matlab-online.html)

*Preprocessing of microscopic images*

Microscopic images of the experiment were pre-processed by imageJ software in batches (Code 1). The images were pre-processed by removing high and low frequency components through Band-pass (Gaussian) filter after removing the single-pixel noise through despeckle filter. [see Supplemental materials in Xu *et al*.^19^ The pre-processed images were then binarized using the Auto Local Threshold plug-in (Sauvola’s local thresholding algorithm). Finally, the binary images will then undergo the Fill holes and Open function to fill the holes detected and to remove single-pixel width features. The result of the remaining objects in the image is then considered to be as cells.

*Quantification of cell alignment*

The orientation distribution of the myoblast cells is determined by implementing the custom-made functions in MATLAB® Online™ as shown below (Code 2). The custom-made function was made to read the processed binarized images and estimated as ellipse and the orientation of the ellipse’s major axis (x-axis) then gives the information of the cell orientation.^20^

The information of the cells was then extracted to Microsoft Excel to generate the cell alignment index. the angles were binned into 19 groups for every 10°. Frequency of each group was expressed relatively to the total number of cells and the total number of cells (frequency) was set as 100%.

**Image J batch (Code 1)**

run("8-bit");

run("Despeckle");

run("Bandpass Filter...", "filter_large=40 filter_small=3 suppress=None tolerance=5 autoscale saturate");

run("Auto Local Threshold", "method=Sauvola radius=15 parameter_1=0 parameter_2=0 white");

run("Fill Holes");

run("Auto Local Threshold", "method=Sauvola radius=15 parameter_1=0 parameter_2=0");

**Matlab (Code 2)**

I = imread()

BWConv = bwconvhull(~I);

I = I & BWConv;

figure()

h = imshow(I);

axis on

stats = regionprops('table',I,'Area','Centroid','MajorAxisLength','Orientation')

nObjects = size(stats,1);

head(stats)

hold on

ph1 = plot(stats.Centroid(:,1), stats.Centroid(:,2), 'rs');

stats.Slope = atan(-stats.Orientation*pi/180);

stats.Intercep = stats.Centroid(:,2) - stats.Slope.*stats.Centroid(:,1);

stats.EndpointX = stats.Centroid(:,1) + [-1,1].* (stats.MajorAxisLength/2 .* sqrt(1./(1+stats.Slope.^2)));

stats.EndpointY = stats.Centroid(:,2) + [-1,1].* (stats.Slope .* stats.MajorAxisLength/2 .* sqrt(1./(1+stats.Slope.^2)));

mah = plot(stats.EndpointX.', stats.EndpointY.', 'r-')

**
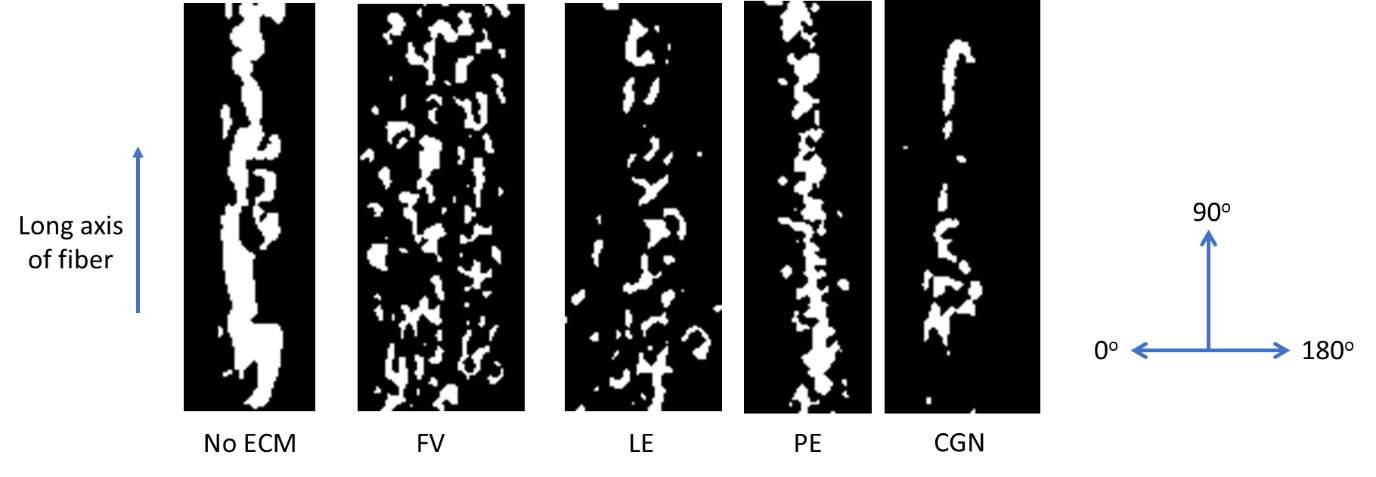
**

Supplementary Figure 3-1. Representative binary images of cells in a length of fiber containing particulate extract from each of the 3 fungal species (FV, LE and PE) compared to the `no ECM’ fiber and collagen (CGN) controls. As the cell-cell interaction in empty fibers are greater than the cell-matrix interactions, the cells tend to aggregate into larger clumps. On the other hand, cell-matrix interactions are greater than cell-cell interactions in fibers containing the fungal extracts and collagen, thus individual cells and smaller clumps of cells are evident.

Thus, while both `no ECM’ fibers and fungal extract-containing fibers have a high frequency of objects at an orientation angle of 90^o^ (along the long axis of the fiber) due to the confinement effect of the fiber, only the fungal extract-containing fibers have a significant number of objects perpendicular to the long axis of the fiber, i.e. at orientation angles of 0^o^ and 180^o^. This occurs because individual cells and smaller clumps of cells that are adjacent to each other in the transverse plane of the fiber form objects that are oriented at angles 0^o^ and 180^o^. (See Figure S3-2)

**
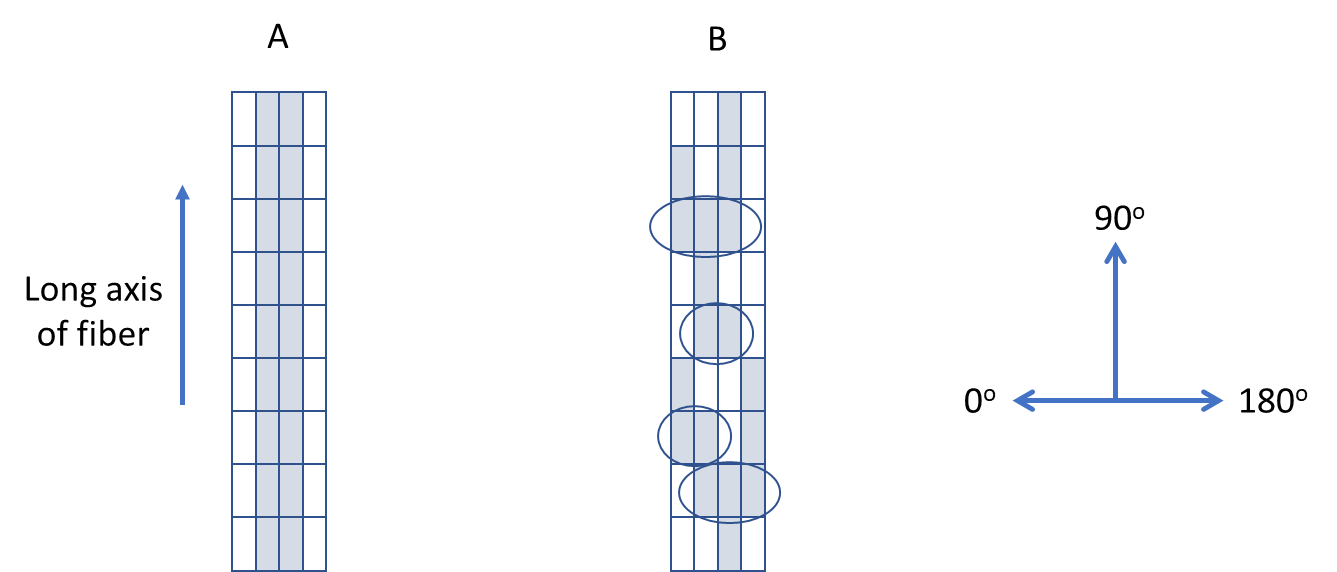
**

Supplementary Figure 3-2. When cell-cell interactions > cell-matrix interactions, a large cluster of cells form which lacks objects oriented at 0^o^ or 180^o^. (B) When cell-matrix interactions > cell-cell interactions, individual cells and smaller clumps of cells are present. When these are adjacent in the transverse plane of the fiber, they form objects (encircled) that are oriented at 0^o^ or 180^o^.

**4. Modelling of Protein Structures**


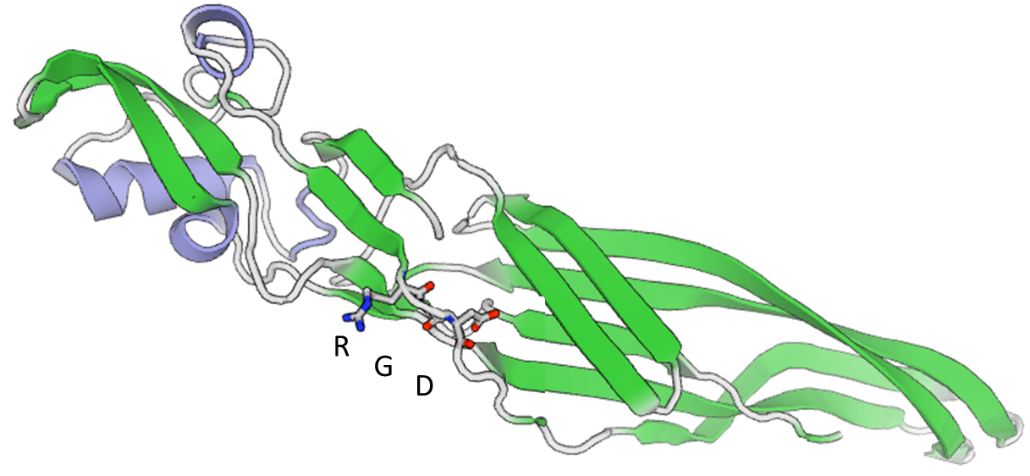


Supplementary Figure 4-1: Modelling of C1 protein structure showing RGD motif present on molecular surface. The protein sequence of C1 protein (Accession ID: O74163) was modelled via SWISS-MODEL ^17^. Crystal structure of the parasporin-2 Bacillus thuringiensis toxin that recognizes cancer cells^18^ (SMTL ID: 2ztb.1) was used as the template for this modelling.

Supplementary Figure 4-2: Surface models obtained from SWISS-MODEL for (A) Beta-glucosidase (Homo-tetramer) (Accession: G8A544), (B) C1 protein (Accession: O74163), (C) Aryl-alcohol oxidase (Accession: O94219), (D) Zb protein (Accession: Q7ZA62), (E) Nucleoside diphosphate kinase (Homo-hexamer) (Accession: A0A1Q3EJT6). RGD motifs are highlighted in gold.

**REFERENCES**

1. Pöhlmann, J. & Fleig, U. Asp1, a Conserved 1/3 Inositol Polyphosphate Kinase, Regulates the Dimorphic Switch in Schizosaccharomyces pombe. Mol Cell Biol 30, 4535–4547 (2010).

2. Lima, M. A. et al. Aspergillus niger β-Glucosidase Has a Cellulase-like Tadpole Molecular Shape. Journal of Biological Chemistry 288, 32991–33005 (2013).

3. Yoshida, E. et al. Role of a PA14 domain in determining substrate specificity of a glycoside hydrolase family 3 β-glucosidase from Kluyveromyces marxianus. Biochemical Journal 431, 39–49 (2010).

4. Kock, M. et al. Structural and Functional Characterization of PA14/Flo5-Like Adhesins From Komagataella pastoris. Front Microbiol 9, (2018).

5. Sigrist CJA, de Castro E, Cerutti L, Cuche BA, Hulo N, Bridge A, Bougueleret L, Xenarios I. New and continuing developments at PROSITE Nucleic Acids Res. 2012; doi: 10.1093/nar/gks1067

6. Várnai, A., Viikari, L., Marjamaa, K. & Siika-aho, M. Adsorption of monocomponent enzymes in enzyme mixture analyzed quantitatively during hydrolysis of lignocellulose substrates. Bioresour Technol 102, 1220–1227 (2011).

7. da Silva, V. M. et al. Non-productive adsorption of bacterial β-glucosidases on lignins is electrostatically modulated and depends on the presence of fibronection type III-like domain. Enzyme Microb Technol 87–88, 1–8 (2016).

8. Blum, M., Chang, H. Y., Chuguransky, S., Grego, T., Kandasaamy, S., Mitchell, A., ... & Finn, R. D. (2021). The InterPro protein families and domains database: 20 years on. Nucleic acids research, 49(D1), D344-D354.

9. MacKenzie, C. R., Hirama, T. & Buckley, J. T. Analysis of Receptor Binding by the Channel-forming Toxin Aerolysin Using Surface Plasmon Resonance. Journal of Biological Chemistry 274, 22604–22609 (1999).

10. Knapp, O. The Aerolysin-Like Toxin Family of Cytolytic, Pore-Forming Toxins. Open Toxinology J 3, 53–68 (2013).

11. Nyanhongo, G. S., Steiner, W., & Gübitz, G. M. (Eds.). (2011). Biofunctionalization of Polymers and their Applications (Vol. 125). Springer Science & Business Media.

12. Dashtban, M., Schraft, H., Syed, T. A. & Qin, W. Fungal biodegradation and enzymatic modification of lignin. Int J Biochem Mol Biol 1, 36–50 (2010).

13. Chanda, K., Mozumder, A. B., Chorei, R., Gogoi, R. K. & Prasad, H. K. A Lignocellulolytic Colletotrichum sp. OH with Broad-Spectrum Tolerance to Lignocellulosic Pretreatment Compounds and Derivatives and the Efficiency to Produce Hydrogen Peroxide and 5-Hydroxymethylfurfural Tolerant Cellulases. Journal of Fungi 7, 785 (2021).

14. Zhang, L. et al. Nucleoside Diphosphate Kinases (ndk) reveals a key role in adhesion and virulence of Aeromonas veronii. Microb Pathog 149, 104577 (2020).

15. Snider, N. T., Altshuler, P. J. & Omary, M. B. Modulation of cytoskeletal dynamics by mammalian nucleoside diphosphate kinase (NDPK) proteins. Naunyn Schmiedebergs Arch Pharmacol 388, 189–197 (2015).

16. Virtanen, P. et al. SciPy 1.0: Fundamental Algorithms for Scientific Computing in Python. Nature Methods 17, 261–272 (2020).

17. Waterhouse, A. et al. SWISS-MODEL: homology modelling of protein structures and complexes. Nucleic Acids Research 46, W296–W303 (2018).

18. Akiba, T. et al. Crystal Structure of the Parasporin-2 Bacillus thuringiensis Toxin That Recognizes Cancer Cells. Journal of Molecular Biology 386, 121–133 (2009).

19. Xu F, Beyazoglu T, Hefner E, Gurkan UA, Demirci U. Automated and adaptable quantification of cellular alignment from microscopic images for tissue engineering applications. Tissue Eng Part C Methods. 2011 Jun;17(6):641-9.

20. Sharma, M. Extracting cell and fibre orientation from microscopic images: computational methods and tools. Current Science. 111, NO. 12, 1936 -1945 (2016).
